# Manufacture of adeno-associated virus vectors by a novel human-derived cell line HAT and comprehensive evaluation of the vectors

**DOI:** 10.1016/j.omta.2026.201700

**Published:** 2026-02-14

**Authors:** Yasuo Tsunaka, Mitsuko Fukuhara, Saki Shimojo, Aoba Matsushita, Takahiro Maruno, Sereirath Soth, Haruka Nishiumi, Mark Allen Vergara Rocafort, Toshie Kuwahara, Kenjiroo Matsumoto, Kosei Shibata, Ryoji Nakatsuka, Ryo Asahina, Saho Mizukado, Yuuki Fukai, Tomoki Togashi, Nemekhbayar Baatartsogt, Kimitoshi Takeda, Atsushi Kuno, Yuji Kashiwakura, Yuki Yamaguchi, Kazuaki Nakamura, Yugo Hirai, Hirokazu Hirai, Tsukasa Ohmori, Takeshi Omasa, Susumu Uchiyama

**Affiliations:** 1 Department of Biotechnology, Graduate School of Engineering, The University of Osaka, 2-1 Yamadaoka, Suita, Osaka 565-0871, Japan; 2 U-Medico Inc., 2-1 Yamadaoka, Suita, Osaka 565-0871, Japan; 3 Chitose Laboratory Corp., 3-2-1 Sakado, Takatsu-ku, Kawasaki, Kanagawa 213-0012, Japan; 4 Molecular and Cellular Glycoproteomics Research Group, Cellular and Molecular Biotechnology Research Institute, National Institute of Advanced Industrial Science and Technology (AIST), 1-1-1 Higashi, Tsukuba, Ibaraki 305-8565, Japan; 5 Department of Neurophysiology & Neural Repair, Gunma University Graduate School of Medicine, Maebashi, Gunma 371-8511, Japan; 6 Department of Biochemistry, Jichi Medical University School of Medicine, 3111-1 Yakushiji, Shimotsuke, Tochigi 329-0498, Japan; 7 Center for Gene Therapy Research, Jichi Medical University, 3111-1 Yakushiji, Shimotsuke, Tochigi 329-0498, Japan; 8 Department of Pharmacology, National Research Institute for Child Health and Development, 2-10-1 Okura, Setagaya-ku, Tokyo 157-8535, Japan; 9 Manufacturing Technology Association of Biologics, 7-1-49 Minatojima-Minamimachi, Chuo-ku, Kobe 650-0047, Japan

**Keywords:** adeno-associated virus, AAV, gene therapy, transduction, serotype, EF ratio, post-translational modification, manufacturing, quality attribute, HAT cell

## Abstract

Recombinant adeno-associated viruses (rAAVs) are prominent vectors in gene therapy. However, efficient, low-cost manufacture of the high-quality rAAVs that are necessary for clinical success remains challenging. Here, we report the manufacture of rAAVs using a novel human-derived suspended cell line, human amniotic epithelial cell line for gene and cell therapy (HAT). The transfection conditions for HAT were optimized for rAAV2, 5, and 9, with their titer and full particle ratio (EF ratio) as critical quality attributes. The EF ratios in HAT cell lysate for rAAV2, rAAV5, and rAAV9 were 46%, 17%, and 76%, respectively, which were all higher than the values from human embryonic kidney 293 (HEK293) cells. After purification of HAT-cell-produced rAAV9 by affinity and anion exchange chromatographies, the EF ratio reached 97%. Culture of rAAV9 in HAT cells in a 2-L bioreactor produced 7.7 × 10^14^ vector genomes/L with an EF ratio of 89%. Comprehensive characterizations of purified rAAVs showed comparable quality attributes between the adeno-associated viruses (AAVs) produced by HAT and HEK293 cells. The *in vitro* and *in vivo* potencies of HAT- and HEK293-cell-produced rAAVs were similar, except for rAAV5, for which the HAT-cell-produced vector had higher potency. This study shows the potential of HAT cells for use in the manufacture of high-quality rAAVs.

## Graphical abstract

**Figure:**
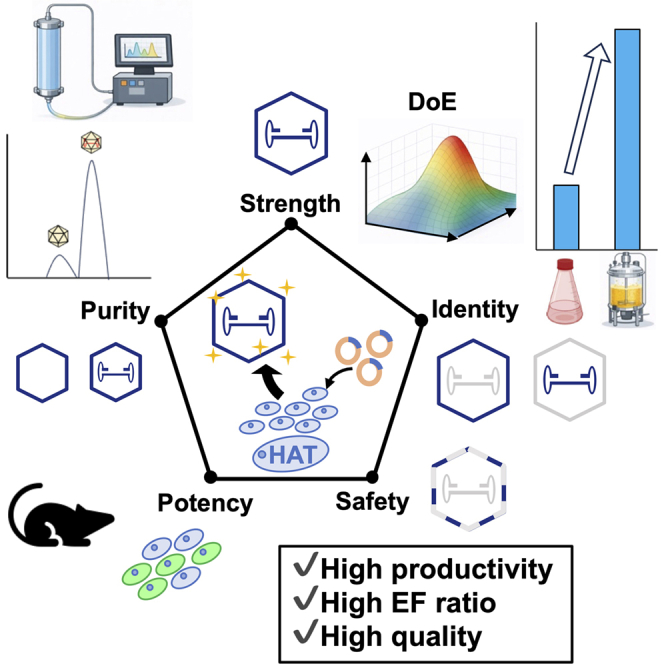

## Teaser

In adeno-associated virus (AAV) manufacturing, HAT cells achieved high productivity with a high EF ratio. Highly purified recombinant AAVs (rAAVs) produced in HAT cells showed quality attributes equivalent to those produced in HEK293 cells. HAT is a high-potential cell line suitable for the production of high-quality rAAVs for clinical trials.

## Introduction

Adeno-associated viruses (AAVs) have three viral capsid proteins: viral protein 1 (VP1), VP2, and VP3.^1^^,^^2^ A viral genome of up to approximately 5 kb is encapsidated.^3^ The virus is approximately 25 nm in diameter with an icosahedral structure.^4^^,^^5^^,^^6^^,^^7^ Recombinant adeno-associated viruses (rAAVs) encapsidating the therapeutic genes are a leading platform for *in vivo* gene therapy because of their wide serotype-dependent tissue tropism, nonpathogenicity, and low immunogenicity compared with other viral vectors.^2^^,^^4^^,^^5^^,^^6^^,^^7^^,^^8^^,^^9^^,^^10^ Eight rAAV therapies have been approved by regulatory agencies,^9^^,^^10^^,^^11^ and many rAAV-based gene therapy trials are ongoing.^12^^,^^13^

The manufacture of rAAVs for clinical use remains challenging because of the complex processes, high costs, and the need for rigorous quality assessment.^14^^,^^15^^,^^16^^,^^17^^,^^18^ Currently, the production of rAAVs is mainly carried out using the triple transfection method (i.e., transfection of three plasmids) into human embryonic kidney 293 (HEK293) cells^19^^,^^20^ or by infection of insect cells with recombinant baculoviruses.^21^^,^^22^ While the baculovirus/Sf9 cell system has achieved high production of rAAVs, this production in a non-mammalian cell line has led to significant differences in critical quality attributes (CQAs) from rAAVs produced in HEK293 cells.^22^^,^^23^ A previous study showed a decrease in the incorporation of VP1 and VP2, an increase in capsid deamidation, and a decrease in the potency of AAV vectors produced in Sf9 cells compared with those produced in HEK293 cells.^21^ Meanwhile, how differences in manufacturing processes affect CQAs are unclear, and the relationships between quality characteristics, efficacy, and safety are still not fully understood.

The triple transfection method was first developed for AAV2 as a scalable AAV production method in suspension culture of HEK293 cells in serum-free medium.^24^ The method has been successfully applied to production of multiple serotypes, from AAV1 to AAV9.^25^ The process, combined with continuous harvesting and anion exchange chromatography (AEX) purification, achieved Good Manufacturing Practice (GMP)-scale, high-quality AAV production using HEK293 cells.^26^ However, the processes based on triple transfection of HEK293 cells result in a low (5%–30%) EF ratio (EF ratio = full particles/[full particles + empty particles]) in the upstream processes.^27^

A low proportion of full capsids in the upstream processes requires the establishment of complex downstream processes with high costs to obtain highly purified AAV,^28^^,^^29^^,^^30^^,^^31^^,^^32^ whereas a high proportion of full capsids in cell culture enables the production of highly purified AAV without the need to improve the purification process.^33^^,^^34^^,^^35^^,^^36^ For example, AAV production using a self-attenuating adenovirus production platform led to a higher EF ratio than that from the triple transfection process, leading to the highly purified AAV through the single AEX chromatography in the polishing process.^35^ Nevertheless, the triple transfection method remains a promising rAAV production platform because of its flexibility and suitability for rapid implementation. Notably, the triple transfection method can be readily applied to novel cell lines as alternatives to HEK293 cells.

HAT cells (human amniotic epithelial cell line for gene and cell therapy), derived from human amniotic epithelial cells, are an immortalized cell line and have been recently established for gene therapy. This is a clonal cell line obtained by single-cell cloning from a cell pool that had been adapted to serum-free suspension in synthetic medium.^37^ The immortalization of HAT cells was conducted by transfecting the cells with a DNA fragment containing the E1 gene region derived from human adenovirus type 5. The E1A and E1B protein expression levels were comparable between adherent HEK293 cells and the HAT cell line.^37^ The HAT cells were prepared from placenta extracted from postpartum women who were confirmed to be negative for hepatitis B, hepatitis C, human immunodeficiency virus infection, adult T cell leukemia, and syphilis.^37^ A recent study demonstrated that HAT cells display high levels of proliferation and outcompete HEK293 cells.^37^ Therefore, it is worth investigating rAAV production by the triple transfection method in HAT cells rather than HEK293 cells.

Design-of-experiment (DoE) methodology is an efficient means of studying the interactions of multiple factors on a specified output and allows users to manipulate several variables simultaneously to determine the optimal value for a given factor. The DoE method has been used successfully to optimize AAV manufacturing processes.^19^^,^^20^^,^^33^^,^^38^^,^^39^^,^^40^^,^^41^ By considering transfection in the triple transfection process, the DoE approach to DNA complexation^39^ and plasmid weight ratios^33^^,^^40^ enabled optimization of both the viral genome titer and EF ratio. DoE optimization of plasmid weight ratios sometimes increased viral genome titers alone.^38^^,^^41^ Using a miniaturized automated bioreactor system, DoE optimization of bioreactor operating and transfection parameters achieved high AAV productivity and a high EF ratio.^33^ Collectively, the triple transfection method combined with DoE methodology is worth applying to rAAV production in HAT cells.

Here, we report the manufacture of rAAV2, 5, and 9, serotypes that have been used in commercially approved pharmaceuticals, by the triple transfection method in the HAT suspended cell system. Enhanced green fluorescent protein (EGFP) was used as the gene of interest (GOI) under the control of the cytomegalovirus (CMV) promoter. The purified HAT-cell-produced AAV vectors were characterized comprehensively and compared with HEK293-cell-produced AAV vectors. Following DoE optimization of transfection conditions, production of the three rAAVs in shaken flasks followed by purification using affinity chromatography and density gradient centrifugation was carried out to prepare highly purified full particles (FPs) for the comprehensive characterizations. Quality attributes (QAs) such as the (VP1 + VP2)/VP stoichiometric ratio, the EF ratio, nucleic acid components, transduction efficiency, and deamidation rates of asparagine residues were determined, and potency assays were performed. The similarity of QAs between the HAT- and HEK-cell-produced rAAVs was also assessed. Our results provide important data for the use of HAT cells for production of high-quality rAAVs for clinical application.

## Results

### Production of rAAVs from different passages of HAT cells

Representative measurements of viable cell concentrations (VCCs) and cell viabilities during culture of suspended HAT cells are shown in Figure S1A up to passage 15 (p15). The VCC for each cell passage started at 0.65 × 10^6^ and 0.25 × 10^6^ cells/mL for 3- and 4-day cultures, respectively. The doubling times ranged from 18 to 22 h (Figure S1B). At passage 5, 10, and 15 (p5, p10, p15), rAAV2 and rAAV9 were produced via the triple transfection method using the transfection reagent FectoVIR-AAV and three plasmids—pGOI, pRepCap, and pHelper. In this experiment, *Zoanthus* green fluorescent protein 1 (ZsGreen1) was used as the GOI under the control of the CMV promoter. Cells harvested at about 72 h post-transfection (HPT) were lysed, and virus genome titers (vector genome [vg]/mL) of the produced rAAVs were quantified via digital polymerase chain reaction (dPCR). rAAVs were stably produced even at p15 (Figure S1C).

### DoE experiments for the optimization of transfection condition

The DoE method was applied to optimize transfection conditions to produce rAAV2, rAAV5, and rAAV9 in the HAT suspended cell system. The triple transfection was performed using three plasmids: pGOI (CMV-EGFP), pRepCap, and pHelper. We selected three input parameters: the ratio of the pGOI and pRepCap plasmid, the ratio of the pGOI and pHelper plasmid, and the ratio of PEI-pro (the transfection reagent) to total DNA. To simultaneously optimize the plasmid ratios and the PEI-pro/DNA ratio, we used response surface methodology, rather than a mixture design (which is typically used for plasmid ratio optimization). Multiple ranges for each parameter were set, as shown in Table 1 (DoE I variation ranges), and 18 experimental transfection conditions were generated (Table S1, runs 1–18). The transfection conditions were tested in 20-mL suspension cultures. Cells harvested at about 72 HPT were lysed, and the amounts of rAAV produced were quantified via quantitative PCR (qPCR), followed by the quantification of the EF ratio using mass photometry (MP) after small-scale affinity purification using AAVX affinity resin.^42^

**Table 1:** Parameter range selected for optimization and DoE-optimized values

Parameter	DoE I Variation range	DoE I Titer-optimized	DoE I EF ratio-optimized	DoE II Variation range	DoE II Titer-optimized	DoE II EF ratio-optimized	DoE II DS-optimized
**HAT/AAV2**							

PEI/DNA	1–2	1.47	<1	1–2	1.32	<1	1
pGOI/pRepCap	0.2–4.5	1.92	3.46	0.2–4.5	0.2	2.65	2.1
pGOI/pHelper	0.5–6.5	<0.5	<0.5	0.1–6.5	<0.1	0.19	0.3

**HAT/AAV5**							

PEI/DNA	1–2	1.96	<1	1–2	1.74	<1	1
pGOI/pRepCap	0.2–4.5	<0.2	2.23	0.2–4.5	1.05	2.94	2
pGOI/pHelper	0.5–6.5	<0.5	<0.5	0.1–6.5	0.72	3.67	2.3

**HAT/AAV9**							

PEI/DNA	1–2	1.47	<1	0.5–2	1.79	<0.5	1.13
pGOI/pRepCap	0.2–4.5	1.92	3.46	0.2–4.5	0.85	2.75	2.3
pGOI/pHelper	0.5–6.5	<0.5	<0.5	0.1–6.5	<0.1	<0.1	0.5

	DoE Variation range	DoE Titer-optimized	DoE EF ratio-optimized	DoE DS-optimized			

**HEK/AAV9**							

PEI/DNA	1–2	<1	<1	1			
pGOI/pRepCap	0.2–4.5	0.3	3.3	2			
pGOI/pHelper	0.5–6.5	<0.5	<0.5	0.5			

**Figure 1:**
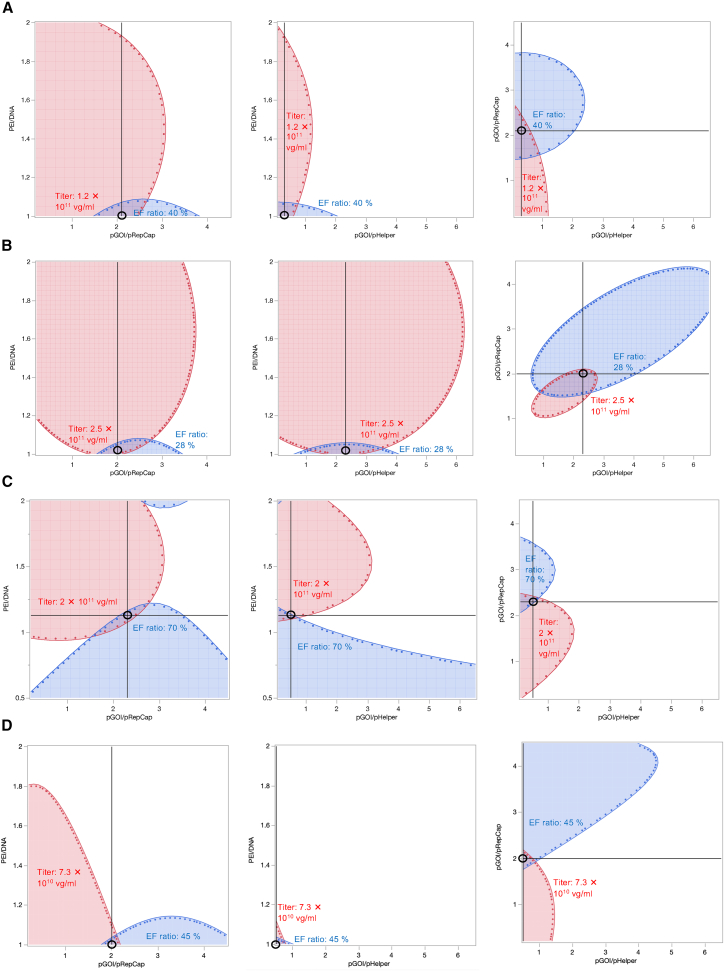
Design space analysis for optimization of rAAV transcription conditions (A–D) Design space (DS) plots showing the probabilities of the rAAV titer and EF ratio of HAT-cell-produced AAV2 (A), AAV5 (B), and AAV9 (C) and HEK293-cell-produced AAV9 (D) in associated parameter ranges. Left, center, and right images show when the ratios of pGOI to pHelper, pGOI to pRepCap, and PEI-pro to DNA were kept at the DS-optimized values, respectively. The red and blue spaces and dots indicate the ranges corresponding to high predicted values of titer and EF ratio, respectively. The selected optimal setpoints are marked with black circles.

The data in Table 1 show that optimization condition in the pGOI/pHelper ratio was predicted out of the parameter ranges in all serotypes. Therefore, we performed an expanded DoE study (DoE II) to identify the optimal conditions. The additional parameters tested in DoE II (Table 1, eight additional conditions; Table S1, runs 19–26) led to the predictions of optimal conditions as shown in Table 1. The design space (DS), visualized as a two-dimensional response grid within the three-dimensional plots (Figures S2 and S3A–S3F), identified conditions where both the titer and EF ratio values were high (Figures 1A–1C). For each serotype, a spot within the overlapping space with high titer and high EF ratio was selected as the DS-optimized condition (Figures 1A–1C; Table 1). Considering the pGOI/pHelper ratio for rAAV9, the predicted titer and EF ratio remained largely unchanged across the range 0.1–0.5 within the overlapping DS. Accordingly, a pGOI/pHelper ratio of 0.5 was selected for subsequent experiments.

**Figure 2:**
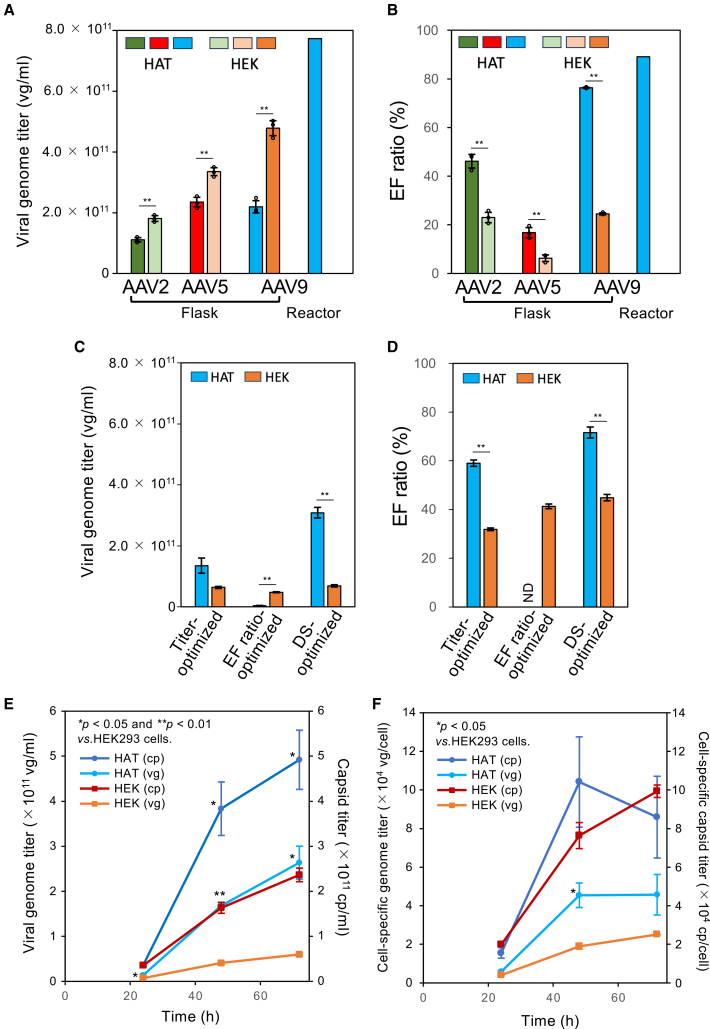
Comparison of rAAV productivity between the HAT and HEK293 cells (A and B) Bar graphs showing the results for HAT cell products in the design-of-experiment (DoE)-optimized conditions and HEK293 cell products in the typical transfection conditions as the genome titer (A) and the EF ratios (B). The genome titers were determined from clarified cell lysates, while the EF ratios were determined from small-scale affinity-purified material. Bars indicating HAT- and HEK293-produced AAV2 are green and light green, respectively; bars indicating HAT- and HEK293-produced AAV5 are red and light red, respectively; bars indicating HAT- and HEK293-produced AAV9 are cyan and orange, respectively. In the flask batches, error bars are ±SD of the mean of triplicate batches per condition. ∗∗*p* < 0.01. The dots show values for each batch. (C and D) Bar graphs showing the results for HAT and HEK293 cell products in the DoE-optimized conditions as the genome titer (C) and the EF ratio (D). Error bars are ±SD of the mean of triplicate batches per condition. ∗∗*p* < 0.01. ND, not detectable due to insufficient capsid titer for MP analysis. (E) Profiles of virus genome titer and capsid titer from HAT cells (genome titer, cyan line and cyan circle plot; capsid titer, blue line and blue circle plot) and HEK293 cells (genome titer, orange line and orange circle plot; capsid titer, deep-red line and deep-red circle plot) over 72 h after transfection in each DoE-optimized condition. Error bars are ±SD of the mean of triplicate batches. ∗*p* < 0.05 and ∗∗*p* < 0.01 *vs.* HEK293 cells. (F) Profiles of cell-specific virus genome titer and cell-specific capsid titer of HAT cells and HEK293 cells over 72 h after transfection in each DoE-optimized condition. Lines and plots use the same color-coding as in (E). Error bars are ±SD of the mean of triplicate batches. ∗*p* < 0.05 vs. HEK293 cells.

Then, the titer and EF ratio obtained under these conditions were verified experimentally using triplicate batches of 200-mL culture of HAT cells. The titer and EF ratio of the HAT-cell-produced rAAVs were compared with those produced using suspended HEK293 cells in which the rAAV production was carried out in the typical transfection conditions^43^ using triplicate batches of the 200-mL culture. The VCC and the cell viability at harvest of the flask batches are summarized in Table S2. Representative MP histograms of HAT- and HEK293-cell-produced rAAVs after small-scale affinity purification are shown in Figures S4A–S4F. For all the three serotypes, the titer of HAT-cell-produced rAAVs was lower than that of the HEK293 cell products (Figure 2A), but the EF ratio was clearly higher than that of the HEK293 cell products (Figure 2B). Surprisingly, the EF ratio of HAT-produced rAAV9 was >75% (Figure S4E), demonstrating noteworthy potential of HAT cells for the production of rAAVs with high EF ratios.

To enable a direct comparison, DoE optimization of rAAV9 production was independently performed in HEK293 cells using the same experimental design space, including identical culture conditions and transfection parameters, as applied to HAT cells. Representative measurements of VCCs and cell viabilities during the culture of HAT and HEK293 cells in identical culture conditions are shown in Figures S5A and S5B. Although the doubling times of the HEK293 cells were slightly decreased compared with those of the HAT cells (Figure S5C), the cell viabilities were comparable (Figures S5A and S5B). For HEK293 cells, each parameter was set similarly to that in HAT cells, and the same 18 conditions were investigated (Table S1, runs 1–18). The genome and capsid titers were determined by qPCR and enzyme-linked immunosorbent assay (ELISA), respectively, using clarified cell lysates. The EF ratios were determined by MP using small-scale affinity-purified materials. We also investigated the capsid titers (capsid particles [cp]/mL) of AAV9 produced in the same 18 conditions using HAT cells by ELISA and compared them between HAT- and HEK293-derived products. As shown in Figure S6A, the capsid titers from HAT and HEK293 cells were comparable in each transfection condition, indicating that capsid production levels were similar between the two cell lines. Notably, both the genome titer and the EF ratio were higher in HAT cells than in HEK293 cells under all conditions (Figures S6B–S6D). These results suggest that the higher titer and EF ratio observed in HAT cells are not simply attributable to differences in capsid production levels but are more likely associated with improved genome packaging efficiency.

The DoE experiments using HEK293 cells led to predictions of the titer-, EF ratio-, and DS-optimized conditions, as shown in Table 1. The DS-optimized condition was identified as a spot within the overlapping space with both a high titer and high EF ratio (Figures 1D and S3G–S3L; Table 1). To validate the predicted optimal conditions, titer-, EF ratio-, and DS-optimized conditions for rAAV9 were tested experimentally using HEK293 cells in triplicate batches of 20-mL culture. We also investigated the titer-optimized and EF ratio-optimized conditions for rAAV9 produced in HAT cells and compared them with the DS-optimized conditions (Figures 2C and 2D). The EF ratio in HAT cells in the EF ratio-optimized condition could not be determined by MP because of low capsid titer and is therefore indicated as not detectable (ND) in Figure 2D. Compared with the titer-optimized and EF ratio-optimized conditions, the DS-optimized conditions showed the comparable or higher values for titer and EF ratio for both HEK293 and HAT cells. In addition, the titer and EF ratio of the HAT cell products in the DS-optimized conditions were significantly higher than those from HEK293 cells. The DoE-optimized conditions for HEK293 cells produced higher EF ratios than the typical transfection conditions, but the titers were lower (Figures 2A–2D, S4F, and S4G).

Measurements of VCCs and cell viabilities of HAT and HEK293 cells during cell culture post-transfection in each DS-optimized condition are shown in Figures S5D and S5E. The HAT cells proliferated from VCC = 1.5×10^6^ cells/mL at transfection, while the proliferation of HEK293 cells was suppressed. The cell viabilities were similar among the cell types. The genome and capsid titers continued to increase in both HAT and HEK293 cells even at 72 HPT, but they were significantly higher in HAT cells than in HEK293 cells (Figure 2E). The cell-specific genome and capsid titers hardly increased from 48 HPT in HAT cells, while the cell-specific genome and capsid titers continued to increase even at 72 HPT in HEK293 cells (Figure 2F).

We then produced rAAV9 using HAT cells in 2-L culture in a 2.6-L bioreactor vessel. The increase in VCC was similar to that in flask batches, while the cell viability was lower than that in the flask batches (Figures S5D and S5E). After cell lysis, the titer was 7.7 × 10^11^ vg/mL (Figure 2A), and the EF ratio was 89% (Figure 2B), both much higher values than were obtained in flask culture. The cell-specific genome titer was 11.4 × 10^4^ vg/cell, which was approximately 2.5 times the mean value of the flask batches (4.6 × 10^4^ vg/cell) (Figure 2F). These results highlight that, in the DoE-optimized transfection conditions used in this study, HAT cells exhibit high rAAV productivity, characterized by both higher titers and higher EF ratios than HEK293 cells.

### Purification of HAT-produced rAAVs

**Figure 3:**
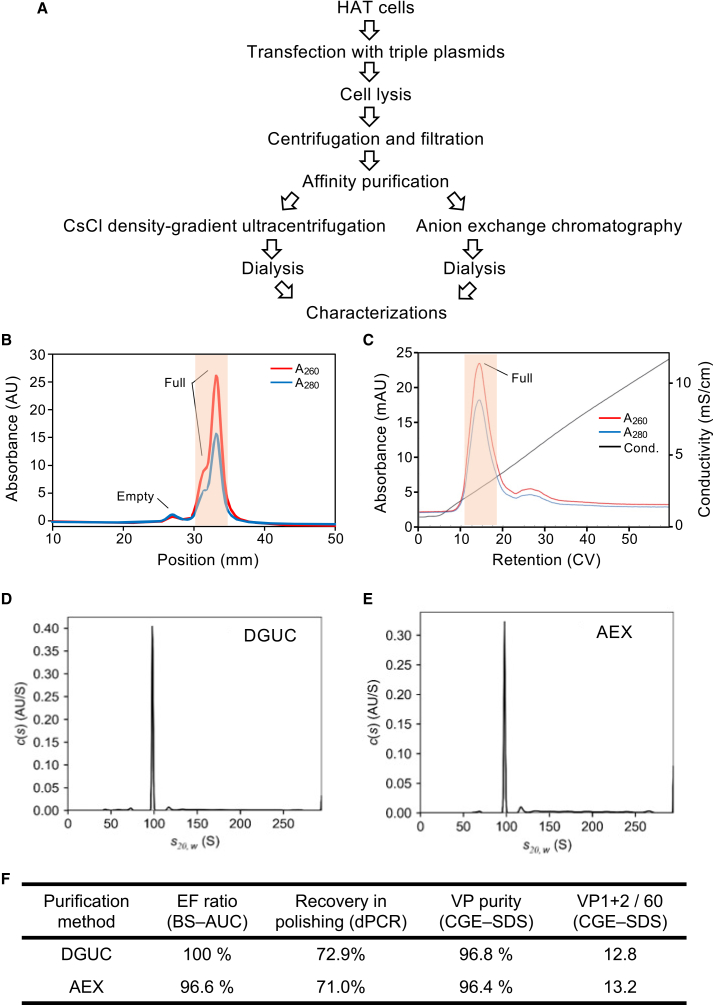
Comparison of different purification methods of HAT-cell-produced AAV9 (A) Scheme of the rAAV production process. (B and C) 280- and 260-nm chromatograms for HAT-cell-produced AAV9 following purification by cesium chloride density-gradient ultracentrifugation (DGUC) (B) or anion exchange chromatography (AEX) (C). The fractions indicated by the orange lines were collected as the purified full particles (FPs). (D and E) Analytical ultracentrifugation sedimentation profile of HAT-cell-produced AAV9 subjected to DGUC (D) or AEX (E) purifications. (F) Table showing quality attributes (QAs) of the DGUC- and AEX-purified AAV9 products.

The rAAV9 produced using HAT cells with the transfection conditions optimized by the DoE approach was purified via the purification processes shown in Figure 3A. Affinity chromatography-purified samples were divided into two portions and then subjected to a polishing process using either AEX or cesium chloride density-gradient ultracentrifugation (DGUC). The purified FP fractions were collected (Figures 3B and 3C) and dialyzed against a formulation buffer. The QAs of the purified rAAV9 products prepared using the two polishing methods were compared.

Size distribution analysis by band sedimentation analytical ultracentrifugation (BS-AUC) showed that the rAAV9 products purified by AEX and DGUC both had an EF ratio of >96% with similar size distribution profiles (Figures 3D and 3E). We also determined the recovery rate from the titers before and after each polishing step, using dPCR. The recovery rate in the DGUC purification was 72.9%, while that in the AEX purification was 71.0% (Figure 3F). These values were almost equal, indicating that the high EF ratio achieved after AEX can be achieved without stringently cutting the collected fractions, as fractions were pooled broadly across the main AEX elution peak (orange line in Figure 3C). The primary nucleic acid and protein components of the rAAV9 products were evaluated by capillary gel electrophoresis (CGE). The encapsidated nucleic acid of the AEX-purified rAAV9 product showed a major sharp peak corresponding to the designed single-stranded DNA (ssDNA), with high similarity to that of the DGUC-purified product (Figures S7E and S7G). The VP purity of both products was >95%, with the number of (VP1 + VP2)/60 being similar for each purification method (Figures 3F, S8A, and S8B). Collectively, the purified rAAV9s had similar QAs regardless of the purification method.

The rAAV2 and rAAV5 produced using HAT cells in the transfection conditions optimized by the DoE approach were purified using the purification process shown in Figure 3A, with polishing carried out by DGUC. For comparison of QAs, the HEK293-cell-produced AAV2, AAV5, and AAV9 (obtained using the typical transfection conditions) were also all purified using the same process. rAAVs with an EF ratio >90% were prepared from all the HAT- and HEK293-produced rAAVs. However, because of the low initial EF ratio of HEK293-produced rAAV5, obtaining an EF ratio of >90% required two rounds of the DGUC purification.

### QAs of HAT-cell-produced rAAVs

**Figure 4:**
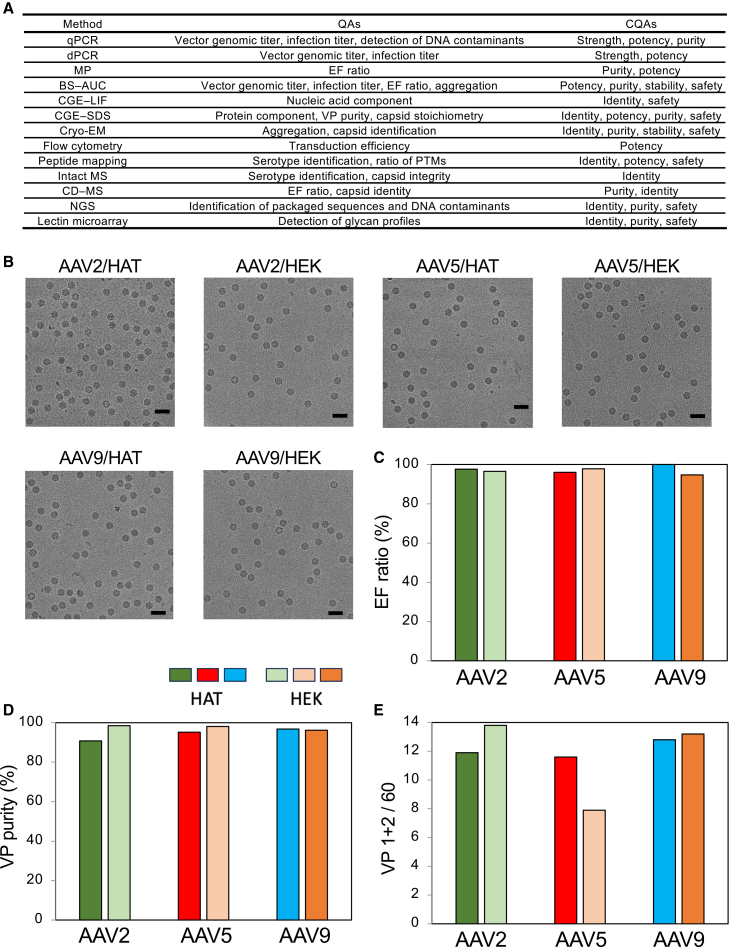
Comparison of QAs between the HAT- and HEK293-cell-derived products (A) Analytical methods used for rAAV characterization in this study. (B) Representative cryo-electron micrographs of the purified AAV2, AAV5, and AAV9 produced using HAT and HEK293 cells. Scale bars, 50 nm. (C–E) Bar graphs showing the analytical results for the purified products derived from HAT and HEK293 cells: EF ratio (C), viral protein (VP) purity (D), and (VP1 + VP2)/60 VP values (E). Bars use the same color-coding as in Figure 2.

The analytical methods used for rAAV characterization in this study are summarized in Figure 4A. The DGUC-purified HAT-cell-produced AAV2, AAV5, and AAV9 were examined by cryogenic electron microscopy (cryo-EM) to evaluate capsid integrity. Cryo-EM micrographs confirmed the formation of rAAV particles with morphologies comparable to those of HEK293-cell-produced AAV2, AAV5, and AAV9 (Figure 4B), consistent with a previous report for AAV8.^44^ Whereas FPs and a few empty particles (EPs) were observed, no assemblies of particles that might have been aggregates were seen.

The EF ratios of the DGUC-purified rAAVs were quantified by BS-AUC; each peak area detected at 230 nm was divided by the molar extinction coefficients at 230 nm (Figures 3D and S9). The results showed EF ratios >95% for purified AAVs produced using HAT and HEK293 cells (Figure 4C).

The encapsidated nucleic acid, evaluated by CGE-laser-induced fluorescence (LIF) (Figures S7A–S7F), showed major sharp peaks corresponding to the designed ssDNA, with high similarity between HAT-produced and HEK293-produced rAAVs. The VP purities of the rAAVs assessed by CGE-sodium dodecyl sulfate (SDS) were >95% for HAT-produced and HEK293-produced rAAVs (Figures 4D and S8). The numbers of VP1, VP2, and VP3 molecules among the 60 VP molecules per AAV vector particle were estimated by dividing the peak areas of VP1, VP2, and VP3 in CGE-SDS by respective molar extinction coefficients at 214 nm. The numbers of (VP1 + VP2)/60 VP for HAT-produced rAAV2 and rAAV9 were equal to those for the HEK293-produced rAAVs, respectively. The number for HAT-produced rAAV5 was higher than that for the HEK293-produced rAAV5 (Figure 4E). Because the numbers of (VP1 + VP2)/60 VP are positively correlated with the transgene expression level of the AAV,^43^^,^^45^ it can be expected that HAT-produced rAAV5 has higher potency than HEK293-produced rAAV5.

For the HAT cell-derived products, the EF ratios and molecular masses of the DGUC-purified rAAVs were determined by charge-detection mass spectrometry (CD-MS). The results showed EF ratios of approximately 100% for the purified AAVs of all serotypes (Figure S10). The molecular mass of each FP was roughly 4.5 M Da.

To rigorously assess the integrity of AAVs produced in HAT cells, we performed the intact mass spectrometry (MS) analyses of the DGUC-purified rAAVs (Table S3). The measured masses of VP1, VP2, and VP3 produced in both HAT and HEK293 cells were equivalent to the theoretical masses. For the calculation of VP1 and VP3 theoretical values, the initial methionine and N-terminal acetylation were removed. These results demonstrate that HAT cells are capable of producing intact AAVs equivalent in quality to those from HEK293 cells.

To test for DNA impurities, we investigated the presence of host-cell DNA (HCDNA) in the purified rAAV9s from HAT and HEK293 cells by qPCR. HCDNA concentrations of the rAAV9 preparations produced in HAT and HEK293 cells were found to be 13.2 and 253.6 ng/mL using the resDNASEQ Human Residual DNA Quantitation Kit and 10.6 and 236.6 ng/mL using the resDNASEQ Quantitative HEK293 DNA Kit, respectively. Therefore, rAAV9 preparations produced in HAT cells contained lower amounts of residual HCDNA than those produced in HEK293 cells.

To identify packaged sequences and DNA contaminants, we investigated the sequences of the AAV genomes from rAAV9s produced in HAT and HEK293 cells using next-generation sequencing. The size distribution of the nanopore sequencing reads and the contamination levels of pHelper, pRepCap, and the plasmid backbone of pGOI were similar between the HEK293- and HAT-cell-derived products (Figure S11). In contrast, the contamination level of HCDNA was lower in the HAT cell product (0.08%) than the HEK293 cell product (0.89%), corresponding to the results for HCDNA detection by qPCR.

**Figure 5:**
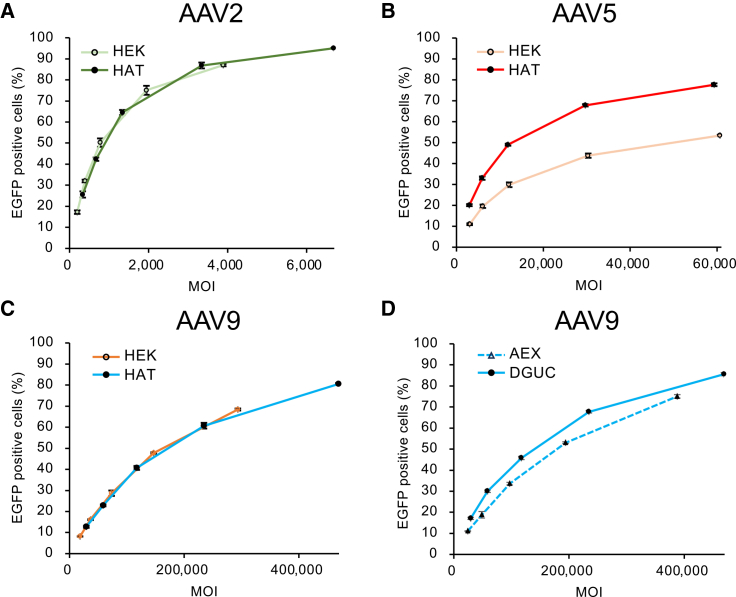
Comparison of transduction efficiency across producing cell lines and rAAV purification methods (A–C) Transduction efficiency comparing the HAT- and HEK293-derived products of AAV2 (A), AAV5 (B), and AAV9 (C). (D) Transduction efficiency comparing DGUC- and AEX-purified HAT-cell-produced AAV9. Lines use the same color-coding as in Figure 2. Error bars are ±SD of the mean of triplicate experiments.

HeLaRC32 cells were then transduced with HEK293- or HAT-produced rAAVs at various multiplicities of infection (MOIs), and the transduction efficiencies were evaluated based on the percentage of EGFP-positive cells (Figure 5). The transduction efficiencies of both HAT-produced rAAV2 and rAAV9 were comparable to those of the HEK293-produced rAAV2 and rAAV9, respectively (Figures 5A and 5C). As expected from the difference in the VP number, the transduction efficiency of HAT-produced rAAV5 was higher than that of HEK293-produced rAAV5 (Figure 5B). To investigate the effect of the different purification process on the transduction efficiency of rAAV9, we also evaluated the transduction efficiencies of HAT-produced rAAV9 purified using AEX or DGUC, respectively. The transduction efficiency of rAAV9 purified by AEX was slightly lower than that of rAAV9 purified by DGUC (Figure 5D). This result is consistent with a previous study, which showed that CsCl exposure during DGUC had no significant impact on transduction efficiency.^46^

### Post-translational modifications of HAT-cell-produced rAAVs

**Figure 6:**
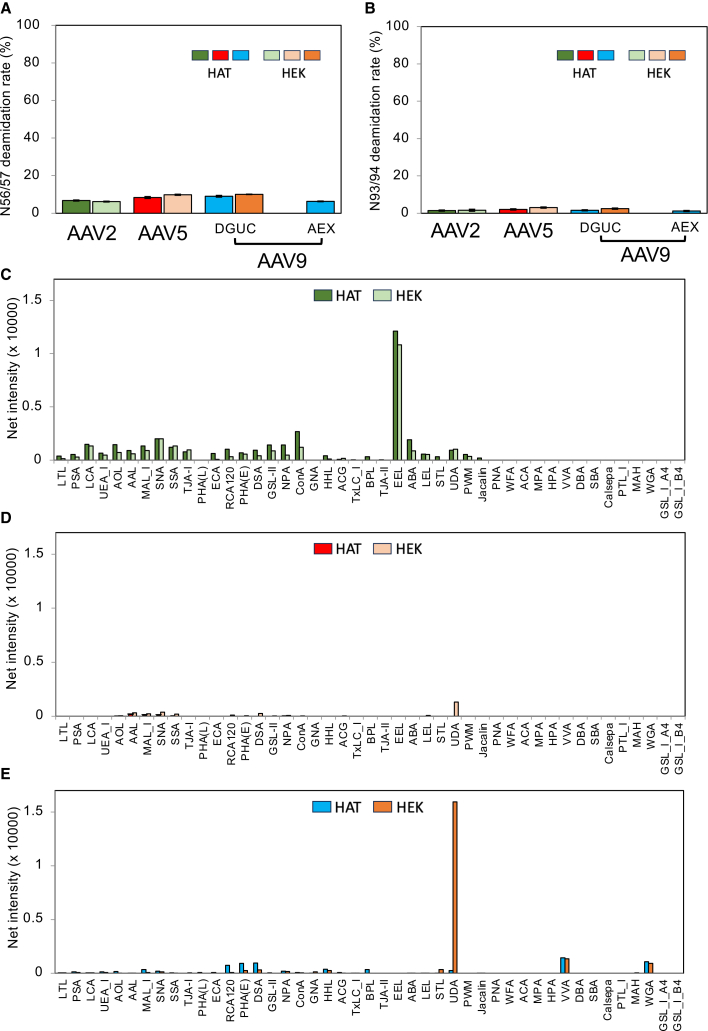
Comparison of post-translational modifications between HAT- and HEK293-cell-derived products (A and B) Bar graphs showing the analytical results for the purified products derived from HAT and HEK293 cells: N56/57 deamination (A) and N93/94 deamidation (B). Error bars are ±SD of the mean of triplicate experiments. (C–E) Lectin microarray analysis comparing HAT- and HEK293-cell-derived products of AAV2 (C), AAV5 (D), and AAV9 (E). The net intensity value for each spot was calculated by subtracting the background value from the signal intensity values of three spots. Bars use the same color-coding as in Figure 2.

The post-translational modifications (PTMs) of HAT- and HEK293-cell-produced rAAVs were examined by liquid chromatography-tandem mass spectrometry (LC-MS/MS). Here, we evaluated the potential deamidation, phosphorylation, and oxidation sites of the capsid proteins in AAV2, AAV5, and AAV9 in triplicate experiments. Of the deamidation sites, amino acid residues N56 (AAV5) or N57 (AAV2 and AAV9) and N93 (AAV5) or N94 (AAV2 and AAV9) had mean deamidation of >1% for all products. Deamidation of these residues, which are conserved among serotypes, has been reported to decrease the transduction efficiency of rAAV8.^47^^,^^48^ In all serotypes, HAT-produced and HEK293-produced rAAVs had similar deamidation rates to each other at N56/57 and N93/94, respectively (Figures 6A and 6B). In rAAV9, deamidation was observed at >1% at non-conserved residues N328, N329, and N452. While there are no reports on the impact of these deamidations on transduction efficiency, deamidation at N329 and N452 can cause immunogenicity.^49^ The deamidation rates were similar between the HAT cell products and the HEK293 cell products (Figure S12A). We also evaluated the deamidation rates of HAT-produced rAAV9 purified using AEX and DGUC; the deamidation rate for rAAV9 was similar irrespective of the purification method (Figures 6A, 6B, and S12A).

The mean S149 phosphorylation rate was >1% for all rAAV2 and rAAV9. The S149 phosphorylation rate was slightly higher in HAT-cell-produced rAAVs than in HEK293-cell-produced rAAVs (Figure S12B). The mean M434 (AAV2) or M436 (AAV9) oxidation rate and the M634 (AAV2) or M635 (AAV5 and AAV9) oxidation rate were >4%. The Met oxidation rates in the HAT-produced AAVs were similar to those in the HEK293-produced AAVs (Figures S12C and S12D).

To investigate potential glycan signatures of HAT-produced rAAVs, we performed antibody-overlay lectin microarray analysis, as established in the previous study,^50^^,^^51^ on the rAAVs produced by HAT and HEK293 cells. As shown in Figures 6C–6E, the lectin array results revealed similar glycan signatures between the HAT- and HEK293-cell-derived samples of each serotype, with a very low population of glycosylation. The weak ConA signal detected in AAV2 is considered to originate from trace contaminant glycoproteins rather than capsid glycosylation, with an estimated glycan content of <0.1%, as reported previously.^52^ This level is substantially lower than that reported for rAAV6 (<2% *O*-glycosylated particles).^51^ Notably, a higher signal was observed for *Urtica dioica* agglutinin (UDA) in HEK293-produced rAAV9 than in HAT-produced rAAV9 (Figure 6E). Previous LC-MS/MS analysis of the HEK293-produced rAAVs identified a UDA peptide derived from the host AAV receptor.^52^ Therefore, it is likely that HAT-cell-produced rAAV9 has a decreased amount of the host AAV receptor, which is an impurity. These results highlight that HAT cells are suitable for rAAV production from the standpoint of glycan quality.

### *In vivo* potency of HAT-cell-produced rAAVs

**Figure 7:**
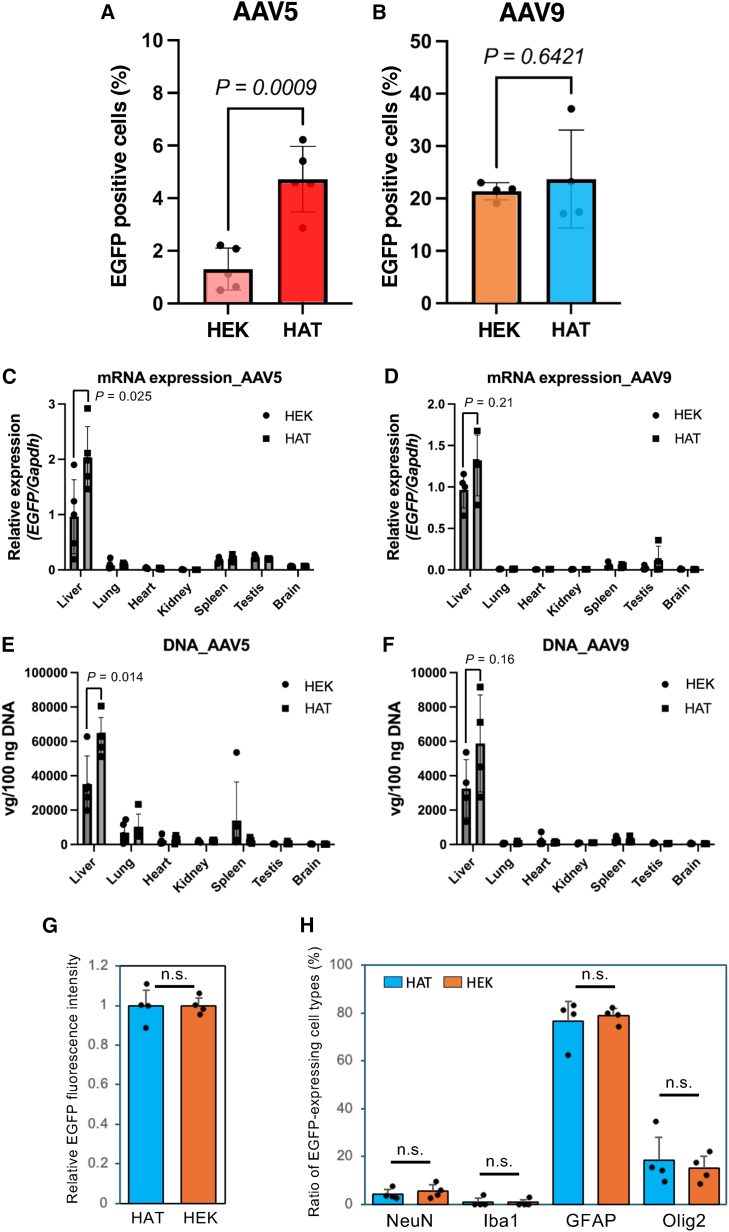
Comparison of murine gene transfer potency between the HAT- and HEK-cell-derived products (A and B) Comparison of *in vivo* gene transfer efficiency of AAV5 (A) and AAV9 (B) derived from HAT and HEK293 cells in mouse liver. Enhanced green fluorescent protein (EGFP) expression in the liver was evaluated using quantitative data for the EGFP-positive cells in immunostaining. (C and D) Comparison of mRNA expression in various mouse organs by the HAT products and HEK293 products of AAV5 (C) and AAV9 (D). A relative quantity of mRNA expression from HEK-cell-produced AAV in liver was set to 1. (E and F) Comparison of genomic DNA in each organ by the HAT products and HEK293 products of AAV5 (E) and AAV9 (F). (G) Comparison of EGFP fluorescence intensity after bilateral injection of the HAT- or HEK-cell-produced rAAV9 into the mouse motor cortex. The average intensity from HEK-cell-produced AAV was set to 1. Each closed circle represents the value from one hemisphere (*n* = 4 hemispheres from two mice). n.s., not significant by unpaired *t* test. Error bars indicate SD of experimental replicates. (H) Proportions of EGFP-positive cell types. The percentages of neurons (NeuN-positive), microglia (Iba1-positive), astrocytes (GFAP-positive), and oligodendrocytes (Olig2-positive only) among EGFP-expressing cells are shown. Each closed circle represents the value from one hemisphere (*n* = 4 hemispheres from two mice). n.s., not significant by unpaired *t* test. Error bars indicate SD of experimental replicates. Bars use the same color-coding as in Figure 2.

To evaluate the efficiency of gene transfer by HAT-produced rAAV *in vivo*, rAAV with the encapsidated gene encoding EGFP under the control of the CMV promoter was administered intravenously to 7-week-old C57BL/6 mice at 1 × 10^11^ vg/body for rAAV5 or 1 × 10^10^ vg/body for rAAV9. After 3 weeks, the mice were sacrificed, and the liver tissue was subjected to immunohistochemical staining (Figure S13), and DNA and RNA were extracted from multiple organs. Consistent with the results of the cell-based assay (Figure 5B), liver transduction efficacy of rAAV5 produced in HAT cells was significantly higher than that of rAAV5 produced in HEK293 cells (Figure 7A). The efficiency of gene transfer of HEK293- and HAT-cell-produced rAAV9 into the liver was equivalent (Figure 7B). Administration of rAAVs resulted in high levels of transgene mRNA expression and vector copy number in the liver (Figures 7C–7F). Notably, both values were significantly higher with HAT-cell-derived rAAV5 than with HEK293-cell-derived rAAV5 (Figures 7C and 7E; mRNA, *p* = 0.025; AAV genome, *p* = 0.014), whereas no significant difference was observed between the two vector sources for rAAV9 (Figures 7D and 7F; mRNA, *p* = 0.21; AAV genome, *p* = 0.16).

rAAV9 (2.5 × 10^11^ vg/mL, 1 μL) was also injected into the motor cortices of 7-week-old C57BL/6 mice, and EGFP expression levels were compared by measuring fluorescence intensity 3 weeks later (*n* = 4 hemispheres). EGFP expression at the cortical surface was equivalent between HEK293- and HAT-cell-produced rAAV9 (Figures 7G and S14A). Also after 3 weeks, sagittal brain sections were prepared and subjected to immunohistochemistry using cell-type-specific markers. Within the EGFP-expressing area (313.75 × 313.75 μm^2^), the densities of neurons, astrocytes, and microglia were quantified. Neuronal loss may indicate degeneration, and increased astrocytes or microglia densities may reflect inflammation. No significant differences in neuronal, astrocytic, or microglial density were observed at the injection site (Figure S14B), indicating no difference in cytotoxicity between HEK293- and HAT-cell-produced rAAV9. The cell types expressing EGFP were identified (Figure S14C), and their proportions were calculated. No significant differences in the proportions of EGFP-positive cell types (NeuN-positive, neurons; Iba1-positive, microglia; GFAP-positive, astrocytes; and Olig2-positive, oligodendrocytes) were observed between the two AAV preparations (Figure 7H).

## Discussion

Previous studies aimed at efficient rAAV production focused on improving the conventional HEK293 host cell line to increase the product yield. Recently, however, a novel host cell line, HAT, tailored to increased rAAV production, was established.^37^ HAT cells are single cloned cells adapted to suspension culture conditions in serum-free medium to allow widespread industrial adoption. Here, with further process development, rAAV production was achieved in HAT cells with scalable production at bioreactor levels (Figure 2), indicating the potential of HAT cells to be a host cell platform that will advance biopharmaceutical manufacturing.

The triple transfection method with DoE optimization of the plasmid ratio was applied in HAT cells and resulted in higher EF ratios than were observed in HEK293 cells under typical transfection conditions (Table 1; Figures 1 and 2). When the same DoE setting was applied to HEK293 cells, similar trends were observed, including a relatively high ratio of pGOI to pRepCap and a higher EF ratio compared with the typical conditions (Table 1; Figure 2). In all previous DoE approaches in HEK293 cells,^33^^,^^38^^,^^40^^,^^41^ a low ratio of pGOI to pRepCap was optimal for rAAV production (Table S4); however, in this study, a relatively high ratio of pGOI to pRepCap was optimal for production in both HAT and HEK293 cells. This discrepancy is likely attributable to differences in experimental parameters, including culture conditions, VCC at transfection, the transfection reagents used, and the serotype, rather than to cell-type-specific effects. In the previous studies,^33^^,^^38^^,^^40^^,^^41^ a high ratio of pGOI to pRepCap resulted in a lack of Rep and Cap proteins, inhibiting capsid packaging and decreasing the genome titer. To prevent this, a low ratio of pGOI to pRepCap was necessary, but this in turn limited the EF ratio. However, in the optimal conditions in this study, efficient rAAV genome packaging was achieved even at a high ratio of pGOI to pRepCap. This suggests that Rep and Cap expression were unlikely to be limiting under these conditions, allowing a high EF ratio, regardless of cell type. Notably, in comparable optimized conditions, HAT cells produced a higher titer and EF ratio of rAAV9 than HEK293 cells, suggesting that a higher ratio of pGOI to pRepCap may be particularly favorable for rAAV production in HAT cells.

For rAAV9, bioreactor culture in DoE-optimized transfection conditions achieved both high productivity (7.7 × 10^14^ vg per 1-L culture) and a high EF ratio (89%) (Figure 2). This productivity of rAAV9 in HAT cells was much higher than that (1.7 × 10^13^ vg per 1-L culture) of rAAV9 in HEK293 cells in a previous study using WAVE bioreactors.^26^ Although direct comparisons across serotypes must be made with caution, we further note that the productivity in this study was somewhat lower than that (1.1 × 10^15^ vg per 1-L culture) of rAAV8 in HEK293 cells using bioreactor culture after optimization of the transfection conditions by DoE^33^; however, the EF ratio (89%) was much higher than that from the HEK293 cells (31.6%). Because these products are applied clinically at doses based on vg content, a high EP content in an rAAV drug exposes patients to higher levels of potentially immunogenic empty capsids without providing any therapeutic benefit.^53^^,^^54^ In a mouse experiment, the transgene expression was repressed by up to 44%–64% by high EP content, while alanine transaminase activity, a marker of liver damage, increased,^55^ suggesting that EPs within the rAAV drug in clinical trials may decrease the *in vivo* potency and increase the risk of liver toxicity. Removing EPs during the manufacturing process may improve the safety and efficacy of rAAV drugs by decreasing the dose that must be administered. However, in the previous study using HEK293 cells, the EF ratio after AEX purification was approximately 60%–80%.^56^ Therefore, it is desirable to produce a high EF ratio at culture. In this regard, production of rAAV by HAT cells has a great advantage for safe gene therapy.

With the aim of achieving a high EF ratio at culture, the self-attenuating adenovirus platform,^35^ the herpes simplex virus platform,^36^ and the AAV producer cells^34^ were previously developed; the platform using adenovirus achieved a high EF ratio (91%) and recovery (71%) of rAAV purified by single AEX chromatography in the polishing process. In comparison with this, HAT cells have superior potential as a cell line for vector production: triple transfection into HAT cells resulted in a higher EF ratio (97%) and the same recovery (71%) following single AEX column purification (Figure 3). The triple transfection method remains a promising rAAV production platform because of its flexibility, ease of rapid implementation, and suitability, especially in the early clinical (trial) stages that require rAAV drug product for a smaller number of patients within tight timelines. Considering the necessity of scaling up rAAV manufacturing in later clinical stages, which is conducted by pharmaceutical companies, AEX is becoming the primary choice for the polishing step to remove EPs; therefore, HAT cells could also be suitable even for the production of rAAVs for late clinical stage needs.

Using the analytical methods shown in Figure 4A, we evaluated size distributions, including the EF ratio, protein components, nucleic acid components, PTMs, and *in vitro* and *in vivo* potencies of HAT-cell-produced rAAVs. The primary aim of process development in AAV manufacturing is to increase the purity and quality of the vectors eventually intended for clinical use.^14^^,^^15^^,^^16^ HAT cells demonstrate the ability to generate higher EF ratios than conventional HEK293 cells while maintaining key QAs such as the deamidation rate, (VP1 + VP2)/VP ratio, and potency.

Sf9 cells also have the ability to generate a higher EF ratio than HEK293 cells. However, rAAV production using Sf9 cells results in lower (VP1 + VP2)/VP ratios, higher deamidation, and differences in PTMs compared with HEK293 cells.^21^ Furthermore, Sf9 cells are prone to contamination with insect-specific host cell proteins (HCPs) and glycans, raising concerns about immunogenicity.^23^ By contrast, HEK293 cells produced products with a higher (VP1 + VP2)/VP ratio, resulting in higher *in vitro*/*in vivo* potency, and HEK293 cells have a predominance of human-origin HCPs and PTMs, making HEK293-cell-derived products highly acceptable as pharmaceuticals. We confirmed here that the AAV products produced in HAT cells showed similar QAs and PTMs to those produced in HEK293 cells, and the *in vitro*/*in vivo* potencies were also similar (Figures 4, 5, 6, and 7). Therefore, it is likely that HAT cells will also be highly acceptable for the production of pharmaceuticals. In addition, oxidation and deamidation of rAAV capsids mainly occur during downstream processing and storage.^57^ In this study, the downstream process (except for the transfection conditions) was performed using the same processes for HAT and HEK293 cells; as a result, there was no significant difference in product oxidation and deamidation (Figures 6 and S12).

In a clinical trial, if 1 × 10^14^ vg/kg of rAAVs is administered to 10 patients weighing 70 kg, 7 × 10^16^ vg is required. Because AAV production in HAT cells in the reactor was 7.7 × 10^14^ vg per liter of culture, approximately 100 L of culture would be required. However, the required culture volume could likely be decreased by increasing the yield.

During AAV production in the DoE-optimized conditions, the VCC of HAT cells continued to increase, while proliferation of HEK293 cells was suppressed; thus, the VCC of HEK293 cells remained lower than that of HAT cells (Figure S5D). The cell viabilities of both HAT and HEK293 cells decreased to a similar extent (Figure S5E). These results indicate that the HAT cells proliferated even during AAV production, whereas HEK293 cell proliferation was suppressed because of cytotoxicity during AAV production.^58^ In the DoE-optimized conditions, although the cell-specific capsid titers at harvest were comparable between the two cell types (Figure 2F), the proliferation of viable HAT cells resulted in a significantly higher capsid titer than that in HEK293 cells (Figure 2E). Similarly, the genome titer was significantly higher in HAT cells than in HEK293 cells (Figure 2E). These observations suggest that, in HAT cells, proliferation of the viable cells contributes to increased overall AAV productivity. In contrast, in the typical transfection conditions, HEK293 cells achieved higher titers than HAT cells in DoE-optimized conditions (Figure 2) but had markedly lower cell viability at harvest (Table S2). This resulted in a higher proportion of dead cells in HEK293 cultures. In HEK293 cultures, the high proportion of dead cells at harvest may reflect cells that had already completed rAAV production and subsequently lost viability. Although this interpretation is speculative, it suggests that cell death dynamics may play an important role in determining overall AAV productivity in these cultures. Consistent with this interpretation, high productivity in the bioreactor cultures of HAT cells was accompanied by lower viability compared with flask batches (Figure S5E). These results suggest that, in HAT cells, both viable cell proliferation and cell death may influence the AAV productivity. A perfusion culture system and medium developments may be effective in improving both cell proliferation and AAV production. Recently, AAV production using HEK293 cells was enhanced by a perfusion culture system in a bioreactor.^59^ The use of a chemically defined medium optimized rAAV production by transiently transfected suspended HEK293 cells.^19^ It is important for future studies to adapt such systems for AAV manufacture using HAT cells.

Collectively, our data show that HAT is a highly suitable cell line with great potential for scaling to large-scale bioreactor production and manufacture of high-quality rAAVs for clinical trials. Future studies are needed to explore the full potential of HAT cells. For cell culture and AAV production using the HAT cell line, optimization of the production system, culture conditions, and medium is essential. Transfection reagents also represent an important parameter for optimization. Previous studies reported a transcriptomic and proteomic analysis of rAAV production processes based on triple plasmid transfection in HEK293 cells, providing valuable insights into host cell responses and factors that affect the productivity.^60^^,^^61^ Performing similar multi-omics analyses of the HAT cell line would be beneficial for systematic optimization of rAAV production. In addition, regulation of Rep and Cap protein expression in HEK293 cells has been shown to significantly improve rAAV productivity and the EF ratio.^62^ This approach may also be applicable to the HAT cell line and could contribute to further optimization of rAAV yield and quality.

## Materials and methods

### Cell culture

HAT suspension cells were cultured in HE400AZ medium (Gmep Incorporated, Fukuoka, Japan). During cell passage, the initial concentrations were set at 0.65 × 10^6^ and 0.25 × 10^6^ cells/mL for 3- and 4-day cultures, respectively. For the suspended HEK293 suspension cell line, Viral Production Cells 2.0 (VPC2.0; Thermo Fisher Scientific, Waltham, MA) were grown in BalanCD HEK293 medium (FUJIFILM Irvine Scientific, Santa Ana, CA) with 6 mM L-glutamine (Fujifilm Wako Pure Chemical Industries, Tokyo, Japan) and were conditioned and cultured in HE400AZ medium in DoE experiments.

### rAAV production

Three plasmids were transfected into cell for rAAV production, and all rAAVs were produced under the same conditions, except for the culture volume, container type, and transfection conditions. DoE batches were prepared as 0.02-L cultures in 0.125-L flasks, after which cell lysis and small-scale affinity purification were performed. Flask batches for DoE validation were prepared as 0.02- and 0.2-L cultures in 0.125- and 1-L flasks. The medium and cell lysates were clarified, and then the rAAVs were purified using the small-scale affinity purification method described further. This process was repeated three times. A reactor batch was prepared as a 2-L culture in a 2.6-L Univessel SU (Sartorius, Goettingen, Germany). As aforementioned, the medium and cell lysates were clarified, with the rAAVs purified using the same small-scale affinity purification method described further. For DoE experiments, pGOI transgene plasmids (CMV-EGFP), pRep2Cap2, pRep2Cap5, and pRep2Cap9 (for AAV serotypes 2, 5, and 9, respectively), and pHelper were cotransfected at an optimized plasmid ratio into suspended HAT or HEK293 cells (1.5 × 10^6^ cells/mL), and the cells were cultured in a flask or bioreactor vessel using a Biostat controller B (Sartorius, Goettingen, Germany). PEI-pro (Sartorius Polyplus, Illkirch, France) was used as the transfection reagent. At approximately 72 HPT, the medium and cell lysates were harvested. The lysates were clarified by centrifugation at 4,000 × *g* for 20 min and filtration through a 0.22-μm polyether sulfone filter (Sartorius, Goettingen, Germany). In the typical transfection conditions of HEK293 cells, the three plasmids were cotransfected into suspended HEK293 cells (2.0 × 10^6^ cells/mL) at a plasmid ratio of 1:1:1, and the cells were cultured as 0.2-L cultures in 1-L flasks. FectoVIR-AAV (Sartorius Polyplus, Illkirch, France) was used as the transfection reagent. At approximately 96 HPT, the medium and cell lysates were harvested and clarified. For confirmation of AAV production from cells passaged different numbers of times, the three plasmids, including a ZsGreen1-expressing pGOI, were cotransfected into suspended HAT cells (2.0 × 10^6^ cells/mL) at a plasmid ratio of 1:1:1, and the cells were grown as 0.02-L cultures in 0.125-L flasks. FectoVIR-AAV was used as the transfection reagent. At approximately 72 HPT, the medium and cell lysates were harvested and clarified.

### rAAV purification

The clarified lysates from the DoE validation batches were loaded onto POROS GoPure AAVX prepacked columns (Thermo Fisher Scientific) equilibrated with 0.15 M NaCl, 20 mM Tris (pH 7.6), and 0.001% (w/v) Poloxamer 188 (P188; BASF, Ludwigshafen, Germany) for AAV5 and AAV9 or 0.5 M NaCl, 20 mM Tris (pH 7.6), and 0.001% P188 for AAV2. After washing with 1 M NaCl, 20 mM Tris (pH 7.6), and 0.001% P188, the AAV was eluted by low-pH elution in 0.1 M citrate (pH 2.0–2.5), 0.4 M L-arginine, and 0.001% P188. The eluate was collected, pooled, and neutralized, before DGUC or AEX.

For DGUC, the purified samples containing 3 or 3.5 M CsCl were centrifuged at 18,000–25,000 rpm in an Optima XE-90 centrifuge using a Beckman SW41Ti rotor (Beckman Coulter, Brea, CA, USA) at 16°C for 24 h. In the case of AEX, the AAVX eluate was diluted to pH 9 and loaded onto a CIMmultus QA HR (2 μm) monolith column (Sartorius BIA Separations, Ajdovščina, Slovenia) equilibrated with 20 mM Tris (pH 9.0), 0.001% P188, and 2 mM MgCl_2_. FP was eluted from the column using a sodium chloride gradient. FP-containing fractions were collected using an online monitoring apparatus and dialyzed against the formulation buffer, 20 mM Tris-HCl (pH 7.4), 100 mM NaCl, 2 mM MgCl_2_, 58.4 mM sucrose, and 0.001% P188, using Slide-A-Lyzer G2 or Slide-A-Lyzer G3 dialysis cassettes (Thermo Fisher Scientific).

### DoE design

All experimental designs and statistical analyses were performed using JMP software version 18.1.0 for Mac OS. To characterize the impact of the ratios of plasmid 1 (pGOI) to plasmid 2 (pRepCap), ratio of plasmid 1 to plasmid 3 (pHelper), and PEI-pro to DNA on the production of rAAVs in suspended HAT cells, and to maximize the titer and EF ratio, two response surface design experiments were performed and statistically analyzed. Student’s *t* tests were performed where appropriate. *p* < 0.05 was considered statistically significant.

### qPCR

AAV samples were diluted 100-fold with nuclease-free water (Thermo Fisher Scientific) containing 0.01% P188 (Sigma-Aldrich, Burlington, MA, USA). Next, non-encapsidated DNA was digested by treatment with DNase I buffer, which included DNase I (Takara, Shiga, Japan) and 0.0075% P188, at 37°C for 30 min. The DNase I was inactivated by adding EDTA (pH 8.0; Nippon Gene, Tokyo, Japan) to a final concentration of 11.4 mM. Then, the samples were heated at 95°C for 10 min to denature the AAV vector capsid. These pretreated samples were diluted 50-fold with nuclease-free water, and the diluted samples (2 μL per sample) were used for PCR (20 μL per reaction). qPCR was performed in triplicate using specific forward and reverse primers (Hokkaido System Science, Hokkaido, Japan) that targeted the inverted terminal repeat (ITR). The reaction mixtures comprised 0.4 μM primers, nuclease-free water, 1× TB Green Premix Ex Taq II, and 1× ROX Reference Dye II (Takara). An in-house positive control was diluted in nuclease-free water from 2 × 10^7^ to 2 × 10^2^ copies/μL in 10-fold increments to generate standard curves for qPCR quantification. qPCR was performed using a QuantStudio3 instrument and a Fast Optical 96-well reaction plate sealed by optical adhesive film (Thermo Fisher Scientific). The qPCR sequence consisted of enzyme activation at 95°C for 2 min, followed by 35 cycles of 95°C for 5 s and 60°C for 30 s. The following primers were used: ITR forward primer, 5′-GGAACCCCTAGTGATGGAGTT-3′; ITR reverse primer, 5′-CGGCCTCAGTGAGCGA-3′.

HCDNA present in AAV samples was quantified by qPCR using the resDNASEQ Human Residual DNA Quantitation Kit (A26366, Thermo Fisher Scientific) and the resDNASEQ Quantitative HEK293 DNA Kit (A46014, Thermo Fisher Scientific). Samples were diluted in DNA LoBind tubes (Eppendorf) with the DNA dilution buffer supplied with the kit to be within the linear range of the standard curve and heated at 95°C for 10 min to disrupt AAV capsids. No DNase treatment was applied before capsid disruption. PCR was prepared according to the manufacturer’s instructions by adding 10 μL of heated sample to a total reaction volume of 30 μL and was performed in triplicate using a QuantStudio 5 Real-Time PCR System (Thermo Fisher Scientific). The PCR cycling conditions were 95°C for 10 min, followed by 40 cycles of 95°C for 15 s and 60°C for 60 s. Human-specific primers and the probe provided in the kit were used as specified by the manufacturer. For the resDNASEQ Human Residual DNA Quantitation Kit, the standard curve was generated using the human genomic DNA supplied with the kit. For the resDNASEQ Quantitative HEK293 DNA Kit, the standard curve was generated using Quantitative HEK293 Genomic DNA (1592106, American Type Culture Collection). Residual HCDNA levels were calculated from Ct values using QuantStudio Design & Analysis Software v.1.5.1 (Thermo Fisher Scientific).

### Small-scale affinity purification

Small-scale affinity purification was conducted as described previously.^42^ POROS CaptureSelect AAVX Affinity Resin (Thermo Fisher Scientific, Waltham, MA, USA) was added to an Acroprep Advance 96-well filter plate (Pall, 8130). An Andrew+ pipetting robot (Waters, Milford, MA, USA) and an Extraction+ solid phase extraction device (Waters), controlled by OneLab Software (Waters), were used to perform automatic resin equilibration three times with 833 μL of 0.15 M NaCl, 20 mM Tris (pH 7.6), and 0.001% P188 (BASF, Ludwigshafen, Germany) for AAV5 and AAV9 or 0.5 M NaCl, 20 mM Tris (pH 7.6), and 0.001% P188 for AAV2; AAV capture with a total of 1,500 μL of sample application; resin washing five times with 500 μL of 1 M NaCl, 20 mM Tris (pH 7.6), and 0.001% P188; AAV elution with 100 μL of 0.1 M citrate (pH 2.5), 0.4 M L-arginine, and 0.001% P188; and neutralization with 50 μL of 1 M Tris-HCl (pH 9.0) (Nippon Gene).

### MP

MP experiments and analyses were conducted using TwoMP and SamuxMP instruments (Refeyn, Oxford, UK) as described previously.^63^ Briefly, several rAAV samples were diluted 10-fold with phosphate-buffered saline (PBS), as necessary to fall within the appropriate range for MP analysis. A precut 2 × 3 culture well gasket (Grace Bio-Labs, Bend, OR, USA) was placed onto coverslips (24 × 50 mm precision; Thorlabs, Newton, NJ, USA). Then, 10 to 18 μL of 1× PBS was loaded into the well created by the gasket, and the focus was automatically adjusted. Purified rAAV was added to the same wells to give a final volume of 20 μL, and the samples were mixed by pipetting. We used samples ranging from capsid titer 1 × 10^9^ to 5 × 10^10^ for MP measurement. After pipetting, a movie was recorded using AcquireMP software version 2.5.0 (Refeyn) for 60 s. The MP movie files were analyzed using DiscoverMP software version 2.5.0 (Refeyn). The molecular mass of each sample was estimated from the MP contrast distribution by applying a contrast-to-mass calibration obtained using apoferritin (Sigma-Aldrich, catalog no. A3660), and AAV8 EP, which is composed only of VP3.^63^ To quantify the EF ratio, the histograms of the mass distributions were peak-fitted with a Gaussian function for EPs and FPs using an in-house Python script.

### ELISA

Capsid titers of rAAV9 were determined using an AAV9 Titration ELISA kit (Progen) following the manufacturer’s instructions. A series of 2-fold dilutions of the kit’s standard viruses were made to generate a capsid standard curve ranging from 2.0 × 10^7^ to 1.30 × 10^9^ cp/mL. rAAV9 vectors were diluted with the supplied assay buffer. All measurements, including those of unknown samples and blanks, were performed in duplicate, and the mean value was used to calculate AAV9 titers. For the assay, 100 μL of each prepared sample was added to a microwell plate and incubated for 1 h at 37°C. The microwell plate was then washed three times with wash buffer. Biotinylated anti-AAV9 antibody was then added, followed by incubation for 1 h at 37°C. After repeating the washing step, streptavidin-horse radish peroxidase conjugate was added and incubated for 1 h at 37°C. Following another wash, ready-to-use tetramethylbenzidine solution was added and incubated for 15 min at room temperature. The color reaction was stopped by adding ready-to-use sulfuric acid solution. Absorbance was then measured at 450 nm using a SpectraMax 3× microplate reader. The readings for each sample were averaged to determine the final titers using a four-parameter logistic (4PL) curve-fitting model. The 4PL standard curve was generated in Microsoft Excel by plotting the subtracted optical density measurements of the serially diluted kit controls against the corresponding rAAV concentrations.

### dPCR

AAV vector-containing samples were added to a solution containing Poloxamer 188, DNase I buffer, and DNase I. The mixtures were incubated at 37°C for 30 min to digest DNA contaminants located outside the AAV vector capsid. DNase I digestion was quenched by adding EDTA (pH 8.0) to a final concentration of 45 mM. Samples were then heated at 95°C for 10 min to denature the AAV vector capsid. The pretreated samples were diluted to the required concentrations with Poloxamer 188 solution. dPCR targeting the ITR was performed using specific forward and reverse primers (900 nM each) and a specific probe (250 nM) labeled with fluorescein amidite (FAM). Reaction mixtures consisted of primers, probe, nuclease-free water, and QuantStudio Absolute Q Digital PCR Master Mix. dPCR was run on a QuantStudio Absolute Q Digital PCR System with a QuantStudio Absolute Q MAP16 Plate (Thermo Fisher Scientific). The thermal protocol included enzyme activation at 94°C for 10 min, followed by 40 cycles of 94°C for 5 s and 60°C for 30 s. The following primers and probe were used: ITR forward primer, 5′-GGAACCCCTAGTGATGGAGTT-3′; ITR reverse primer, 5′-CGGCCTCAGTGAGCGA-3′; and ITR probe, 5′-[FAM]-CACTCCCTCTCTGCGCGCTCG-[BHQ1]-3′.

### BS-AUC

BS-AUC experiments and analyses were conducted as described previously.^64^ Briefly, 15 μL of buffer or sample (approximately 5 × 10^12^ vg/mL) was loaded into the reference or sample well of a 12-mm band-forming centerpiece (Spin Analytical, South Berwick, ME, USA) with sapphire windows. Subsequently, 250 μL (reference) or 240 μL (sample) of PBS/D_2_O containing 0.001% P188 (Sigma-Aldrich, Burlington, MA, USA) was loaded into the corresponding sector. Sedimentation profiles were acquired at 20°C on an ultracentrifuge (Optima AUC, Beckman Coulter) operated at 20,000 rpm with UV detection at 230 nm. Data were collected every 150 s at 10-μm radial increments. Sedimentation data were analyzed using the analytical zone centrifugation c(s) model in the SEDFIT program (version 14.6e).^65^ The lamella width, frictional ratio, meniscus position, and both time-independent and radial-invariant noise were fitted. The regularization level was set to 0.68. Distributions were evaluated over an s-value range of 0–175 S with resolution 350. Buffer density and viscosity were determined with SEDNTERP.^66^ The apparent sedimentation coefficient was converted to the sedimentation coefficient in water at 20°C (*s*_20,w_) using these values together with the partial specific volume of the FP. The molar extinction coefficients of FPs and EPs were calculated according to the procedure reported in a previous study.^67^ Particle concentrations were calculated from the areas of the FP and EP peaks using their molar extinction coefficients, and the EF ratio was calculated as F/(E + F). Mean and standard deviations of *s*_20,w_ values and EF ratios were determined from three independent experiments.

### CGE-SDS

AAV vector (1 × 10^11^ vg) was mixed with 14.4 μL of 10% SDS (Nacalai Tesque, Kyoto, Japan) and 4.8 μL of 99% 2-mercaptoethanol (Fujifilm Wako Pure Chemical Industries, Osaka, Japan) to a final volume of 80 μL, followed by incubation at 70°C for 3 min. Buffer exchange was performed twice at 14,000 × *g* for 10 min at 20°C using Amicon centrifugal filters (Merck Millipore, Burlington, MA, USA) with a matrix-exchange solution containing 3.5% 2-mercaptoethanol and 0.033% SDS. The resulting samples were again incubated at 70°C for 3 min, followed by adding a 10-kDa internal standard (SCIEX, Framingham, MA, USA) and Milli-Q water to bring the final volume to 70 μL. Capillary electrophoresis was performed on a PA800 Plus Pharmaceutical Analysis system (SCIEX) equipped with a photodiode-array detector (214 nm) and controlled by 32 Karat software (version 10.3). Separations were carried out in bare-fused silica capillaries (50 μm i.d., 30 cm total length, 20 cm effective length; SCIEX). Data acquisition and analysis were conducted with 32 Karat software. After the area of each VP peak was corrected using the extinction coefficient at 214 nm, the stoichiometric ratio of VP molecules within the 60-mer capsid was calculated. Means and standard deviations of VP purity and the stoichiometric ratios of VP molecules within the 60-mer capsid were determined from triplicate experiments.

### CGE-LIF

Nucleic acids were extracted from each sample using Proteinase K (QIAGEN, Venlo, the Netherlands), followed by collecting the extracted nucleic acids using a QIAquick PCR Purification Kit (QIAGEN). An aliquot of the collected nucleic acids was analyzed using a PA800 Plus system (SCIEX) equipped with a laser-induced fluorescence detector. Fluorescence signals were acquired with an excitation wavelength of 488 nm and an emission wavelength of 520 nm. Separations were carried out using bare-fused silica capillaries (50 μm i.d., 30 cm length, 20 cm effective length; SCIEX) and a Nucleic Acid Extended Range Gel (SCIEX) containing SYBR Green II RNA Gel Stain (SCIEX). Electropherogram data were processed and analyzed using 32 Karat Software (version 10.3).

### Cryo-EM

Cryo-EM experiments were conducted as described previously.^44^ Briefly, holey carbon grids (Quantifoil Ultra-Cu R1.2/1.3 300 mesh) coated with carbon film were used to prepare the amorphous specimens. The grids were glow-discharged for 30 s at 15 mA using a VES-10 multicoating unit (Vacuum Device, Ibaraki, Japan) before sample application. Sample solution (3 μL) was applied on the grids for vitrification, performed using a Vitrobot Mark IV System (Thermo Fisher Scientific). All micrographs were acquired using a Tundra Cryo-TEM (Thermo Fisher Scientific) at liquid nitrogen temperature with a 100 keV accelerating voltage under the following measurement conditions: automated acquisition mode, 110,000× nominal magnification, and 4-μm defocusing.

### CD-MS

The experimental workflow largely followed our previous study.^68^ Samples (20 μL) were first diluted and desalted into 200 mM ammonium acetate using a Micro Bio-Spin 6 column (Bio-Rad, Hercules, CA, USA). From the exchanged solution, 5 μL was loaded into a custom-made gold-coated nanocapillary and introduced via the native MS interface of an Orbitrap Q Exactive UHMR (Thermo Fisher Scientific, Bremen, Germany). Electrospray was generated at 1.0–1.3 kV, with the capillary temperature held at 320°C. Inside the instrument’s vacuum region, an in-source trapping voltage of −15 V was applied. A trapping-gas setting of 1–2 with SF_6_ produced a pressure of 2.0 × 10^−8^ to 3.0 × 10^−8^ Pa. Spectra were recorded at 50 k resolving power, and ion flux was controlled by manually setting the injection time to 500 ms. The “detector *m/z* optimization” and “ion-transfer target *m/z*” parameters were both set to high. Each CD-MS measurement lasted 30 min, and raw data were processed using STORIBoard (Proteinaceous, Chicago, IL, USA).

### Next-generation sequencing

Nucleic acids were extracted from each sample using Proteinase K (QIAGEN, Venlo, the Netherlands) and then collected using a QIAquick PCR Purification Kit (QIAGEN). The amount of extracted nucleic acid was quantified using a Qubit fluorometer (Thermo Fisher Scientific). The extracted nucleic acids were subjected to nanopore long-read sequencing, performed by BIKEN Biomics Inc. (Osaka, Japan). Sequencing data from AAVs were analyzed as previously described.^69^ Briefly, nanopore raw sequencing reads were filtered using nanoq (quality score ≥9, read length ≥200 nt) and first aligned with minimap2 to vector and plasmid-derived reference sequences. Reads that did not map to vector references were subsequently aligned to the human genome (GRCh38) to assess HCDNA-derived impurities. Read length and mapping distributions were quantified using samtools and custom Python scripts.

### Flow cytometry

HeLaRC32 cells were seeded at 5 × 10^4^ cells/well in 24-well plates in 0.5 mL of Dulbecco’s modified Eagle’s medium (Sigma-Aldrich) containing 10% fetal bovine serum (HyClone, Marlborough, MA, USA) and 1% penicillin-streptomycin (Fujifilm Wako Pure Chemical). We infected cells with AAV vectors at MOIs of 2.0 × 10^2^, 3.9 × 10^2^, 7.8 × 10^2^, 1.9 × 10^3^, and 3.9 × 10^3^ for HEK-AAV2; 3.3 × 10^2^, 6.7 × 10^2^, 1.3 × 10^3^, 3.3 × 10^3^, and 6.7 × 10^3^ for HAT-AAV2; 3.0 × 10^3^, 6.1 × 10^3^, 1.2 × 10^4^, 3.0 × 10^4^, and 6.1 × 10^4^ for HEK-AAV5; 3.0 × 10^3^, 5.9 × 10^3^, 1.2 × 10^4^, 3.0 × 10^4^, and 5.9 × 10^4^ for HAT-AAV5; 1.8 × 10^4^, 3.7 × 10^4^, 7.3 × 10^4^, 1.5 × 10^5^, and 2.9 × 10^5^ for HEK-AAV9; 2.9 × 10^4^, 5.9 × 10^4^, 1.2 × 10^5^, 2.3 × 10^5^, and 4.7 × 10^5^ for HAT-AAV9 (DGUC purified); and 2.4 × 10^4^, 4.8 × 10^4^, 9.7 × 10^4^, 1.9 × 10^5^, and 3.9 × 10^5^ for HAT-AAV9 (AEX purified), in triplicate. Each MOI was based on FP concentrations calculated from BS-AUC experiments. Cells were incubated at 37°C for 2 days and then harvested. The percentage of viable cells expressing EGFP was determined using the CytoFLEX flow cytometry system (Beckman Coulter).

### Peptide mapping

Sample preparation for the peptide mapping method was described previously.^2^^,^^70^ Capsids of AAV vector sample (4.2×10^11^) were incubated with 50 mM ammonium bicarbonate (Fujifilm Wako Pure Chemical), 6 M guanidine hydrochloride (Sigma-Aldrich), 5 mM Tris (2-carboxyethyl) phosphine hydrochloride (Fujifilm Wako Pure Chemical), and 25 mM iodoacetic acid (Sigma-Aldrich) at 20°C for 1 h in the dark. Proteins were subsequently purified by methanol-chloroform precipitation; four volumes of methanol (Fujifilm Wako Pure Chemical), one volume of chloroform (Fujifilm Wako Pure Chemical), and three volumes of Milli-Q water were sequentially added to the sample with a short vortex-mixing and spin-down following each addition. After centrifugation (10 min, 18,000 × *g*, 4°C), the upper phase was carefully removed and discarded. Three volumes of cold methanol were then added to the lower phase, followed by brief vortex mixing and centrifugation (10 min, 18,000 × *g*, 4°C). The resulting supernatant was discarded, and the pellet was air-dried at room temperature (RT) for 5 min. The pellet was dissolved in 30 μL of 50 mM ammonium acetate containing 20 mM methionine (Sigma-Aldrich) at pH 6.5. RapiZyme trypsin (Waters) and lysyl endopeptidase R (Lys-C; Fujifilm Wako Pure Chemical) were added at a volume ratio of 2:1:15 (RapiZyme:Lys-C:AAV) and incubated at 37°C for 2 h. The digestion reaction was terminated by the addition of 3 μL of 1% (v/v) trifluoroacetic acid (Fujifilm Wako Pure Chemical).

Peptide sample solution (10 μL) was injected, and peptides were separated on an ACQUITY UPLC Peptide CSH C18 column (186005294, Waters) using a Vanquish ultra-high-performance liquid chromatography (UHPLC) system (Thermo Fisher Scientific) with a 45-min binary gradient consisting of solvent A (0.1% [v/v] formic acid in water) and solvent B (0.1% [v/v] formic acid in acetonitrile). The gradient was run from 5% to 35% solvent B at a constant flow rate of 50 μL/min, with the column maintained at 45°C. Peptides were detected using a Q Exactive Plus mass spectrometer (Thermo Fisher Scientific). The higher energy collisional dissociation-tandem mass spectrometry (HCD-MS/MS) method was used, and the top 10 precursor ions were analyzed by MS.^2^ The following parameters were used for MS1 analysis: resolution, 70,000; automatic gain control (AGC) target, 3 × 10^6^; maximum injection time, 100 ms; and scan range, *m/z* 300–2,000. For MS2 analysis, the parameters were as follows: resolution, 17,500; normalized collision energy, 28%; AGC target, 1 × 10^5^; maximum injection time, 200 ms; loop count, 10; TopN, 10; isolation window, 2.0 *m/z*; and dynamic exclusion, 7 s. BioPharma Finder software version 5.0 and Byonic within PMI-Byos version 5.9. 121 × 64 (Protein Metrics) as a node were used to identify peptide spectral matches.

### Intact MS

Sample preparation for the intact mass spectrometry method was performed as previously described.^2^ Capsids of AAV vector samples (4.45 × 10^11^) were denatured at RT for 15 min in 10% acetic acid (Fujifilm Wako Pure Chemical). The samples were then centrifuged at 15,000 × *g* for 10 min, and the supernatant was injected into the mass spectrometer.

VP1, VP2, and VP3 proteins were separated as follows: AAV2 and AAV9 were analyzed using a C4 column (Waters, 186004497), while AAV5 was analyzed using a YMC C18 column (YMC, TA30SP9-15Q1PTC). All separations were performed on a Vanquish UHPLC system with solvent A (0.1% [v/v] difluoroacetic acid in water) and solvent B (0.1% [v/v] difluoroacetic acid in acetonitrile); MS grade difluoroacetic acid (Waters) was used. For AAV2 and AAV9, VPs were separated using a 15-min gradient from 32% to 36% solvent B at a constant flow rate of 200 μL/min, with the column maintained at 80°C. For AAV5, a 23.5-min gradient from 32% to 36% solvent B was used at the same flow rate and temperature conditions. Mass spectrometer analysis of VPs was performed as follows: VPs of AAV2 and AAV9 were detected using a Q Exactive Plus mass spectrometer operated in full MS mode. The following parameters were used for the Q Exactive Plus analysis: scan range, *m/z* 900–6,000; in-source CID energy, 10.0 eV; resolution, 17,500; AGC target, 1 × 10^6^; maximum injection time, 200 ms; and S-lens RF level, 100. VPs of AAV5 were detected using a mass spectrometer (Exploris 240) operated in full MS mode. The following parameters were used for the mass spectrometer analysis: scan range, *m/z* 700–4,000; in-source CID energy, 20 V; resolution, 30,000; normalized AGC target, 300%; maximum injection time, 200 ms; and S-lens RF level, 80. BioPharma Finder software version 5.0 was used for spectral deconvolution.

### Differential glycan profiling using antibody-overlay lectin microarray

Differential glycan profiling of rAAVs was performed using the antibody-overlay lectin microarray method,^50^ which we previously optimized for rAAVs.^51^^,^^52^ The amount of purified rAAVs was adjusted by semi-quantification using chemiluminescent intensities of the VP3 band in western blotting. An appropriate amount of each rAAV was diluted to 60 μL with 1% Triton X-100 in Tris-buffered saline (TBSTx) and then applied to a LecChip 45-uni (Precision System Science Ltd., Chiba, Japan), which comprises seven arrays with triplicated spots of 45 different lectins. After incubation at 20°C for 16 h with gentle shaking, 20 μg of human serum polyclonal IgG (10 mg/mL; Sigma-Aldrich) was added to each array and incubated for 30 min. The reaction solution was discarded, and the glass slide was washed three times with TBSTx. Next, 60 μL of CaptureSelect Biotin Anti-AAVX Conjugate (100 ng; Thermo Fisher Scientific) solution in TBSTx containing 20 μg of human serum polyclonal IgG was applied to the array and incubated at 20°C for 1 h. For rAAV9, biotinylated anti-AAV9 antibody (100 ng; PROGEN Biotechnik GmbH, Heidelberg, Germany) was used instead of CaptureSelect Biotin Anti-AAVX Conjugate. After washing three times with TBSTx, 60 μL of Cy3-labeled streptavidin (200 ng; Merck Millipore) solution in TBSTx was added to the array and incubated at 20°C for 30 min. The glass slide was then rinsed with TBSTx and scanned using an evanescent-field fluorescence scanner, GlycoStationReader2300 (emukk LLC, Mie, Japan). All data were analyzed with GlycoStationToolsPro software version 2.0 (emukk LLC). The net intensity value for each spot was calculated by subtracting the background value from the signal intensity values of three spots. For rAAV9, additional background subtraction was performed using negative control data because there was a significant signal from the detection antibody. Data obtained under suitable time exposure conditions (7 s) that were determined using the glycan profile of a control sample rAAV6, as previously reported,^51^ were used to generate glycan profiles.

### *In vivo* potency assay for liver transduction by intravenous AAV injection

These animal experiments were approved by the Institutional Animal Care and Concern Committee of Jichi Medical University and were conducted in accordance with the committee’s guidelines and the ARRIVE guidelines. Seven-week-old C57BL/6 male mice (Japan CLEA) were intravenously administrated with the AAV vector (1 × 10^11^ vg/body for rAAV5 or 1 × 10^10^ vg/body for rAAV9) harboring EGFP driven by the CMV promoter into the jugular vein, using a 29G micro-syringe (Terumo, Tokyo, Japan). After 21 days, the mice were euthanized under isoflurane anesthesia and perfused with PBS. Genomic DNA from various organs was extracted using an automated nucleic acid extraction system (GENE PREP STAR PI-480, Kurabo), and the amount of AAV vector was quantified by qPCR targeting EGFP. Total RNA was extracted using an RNeasy Mini Kit (QIAGEN), reverse-transcribed using a PrimeScript RT Reagent Kit (Takara), and *EGFP* expression was evaluated relative to *Gapdh* (Mm99999915-g1, Thermo Fisher Scientific) by qPCR. The following primers were used for *EGFP*: probe, 5′-/56-FAM/AAG TTC AGC/ZEN/GTG TCC GGC GA/3IABkFQ/3′; forward primer, 5′-CTG GAC GGC GAC GTA AAC-3′; and reverse primer, 5′-CGG TGG TGC AGA TGA ACT T-3′. Protein expression was analyzed by immunostaining, as described previously.^71^ Briefly, livers were fixed in 4% paraformaldehyde, cryoprotected in sucrose, embedded in OCT, and sectioned at 10 μm. Sections were blocked and stained with anti-GFP and anti-CD146 antibodies, followed by Alexa Fluor-conjugated secondary antibodies. Nuclei were counterstained with 4′,6-diamidino-2-phenylindole (DAPI), and EGFP-positive cells were imaged and quantified by fluorescence microscopy (BZ-X810, Keyence).

### *In vivo* potency assay following bilateral AAV injection into the motor cortices

These animal experiments were approved by the Institutional Animal Care and Use Committee of Gunma University and conducted in accordance with institutional and ARRIVE guidelines. AAV vectors (2.5 × 10^11^ vg/mL, 1 μL) were injected into the bilateral primary motor cortex (M1) of 7-week-old C57BL/6 mice (AP +1.0 mm, ML ±1.5 mm, DV +0.8 mm) over 20 min. Three weeks after AAV injection, the mice were perfused with 4% paraformaldehyde, and whole brains were harvested. EGFP fluorescence in the bilateral motor cortices was imaged using a fluorescence stereo microscope (VB-7010; Keyence, exposure time 3 s). The average fluorescence intensity within the thresholded EGFP-positive area was quantified using Fiji software (https://fiji.sc/). Sagittal brain sections (50 μm) were prepared using a vibratome (VT1200S; Leica, Wetzlar, Germany). Free-floating sections were incubated at RT for 30 min in blocking solution containing 5% normal donkey serum, 0.5% Triton X-100, and 0.05% sodium azide in PBS. Sections were incubated overnight at 4°C with primary antibodies diluted in blocking solution, with gentle shaking. One set was stained for EGFP, NeuN (a neuronal marker), and Iba1 (a microglial marker) and another for EGFP, GFAP (astrocyte marker), and Olig2 (oligodendrocyte marker). Primary antibodies used were rat anti-GFP (Nacalai Tesque), mouse anti-NeuN (Merck, Darmstadt, Germany), rabbit anti-Iba1 (Fujifilm Wako Chemicals), and mouse anti-GFAP (Merck). After two washes in 0.5% Triton X-100 and three washes in 0.1% Triton X-100, sections were incubated for 3 h at RT with fluorophore-conjugated secondary antibodies diluted in blocking solution: Alexa Fluor 488-conjugated donkey anti-rat IgG (Invitrogen, Waltham, MA, USA), Alexa Fluor 555-conjugated donkey anti-mouse IgG (Invitrogen), and Alexa Fluor 647-conjugated donkey anti-rabbit IgG (Invitrogen). Following two washes in 0.5% Triton X-100, three washes in 0.1% Triton X-100, and two washes in PBS, sections were mounted using ProLong Diamond (Thermo Fisher Scientific).

### Statistical analyses

Data are presented as the mean ± SD from three independent experiments unless otherwise stated. For certain assays, technical replicates were performed as indicated in the figure legends. Statistical analyses were performed using an unpaired, two-tailed Welch’s *t* test for pairwise comparisons. *p* < 0.05 was considered statistically significant.

## Data and code availability

The data from this study are available from the corresponding author upon reasonable request.

## Acknowledgments

This study was supported by Grants-in-Aid from “Research and Development of Core Technologies for Gene and Cell Therapy” supported by the 10.13039/100009619Japan Agency for Medical Research and Development (AMED) (grant nos. JP18ae0201001, JP18ae0201002, and JP24se0123004h0101). We thank BASF for providing the Poloxamer 188. We thank Karin Bandoh and Rikako Uemura (The University of Osaka, Osaka, Japan) for the MP, CGE-LIF, and CGE-SDS experiments. We thank Mika Kishimoto, Tamaki Aoki, and Mai Hayashi (Jichi Medical University, Tochigi, Japan) for qPCR, immunostaining, and section preparation in animal experiments. We thank BIKEN Biomics Inc. (Osaka, Japan) for nanopore long-read sequencing. We thank Edanz (https://jp.edanz.com/ac) for editing a draft of this manuscript.

## Author contributions

Conceptualization, S.U.; sample preparation and DoE experiments, Y.T., M.F., A.M., S. Soth, R.A., and Y.H.; methodology, Y.T., M.F., S. Shimojo, A.M., T.M., H.N., T.K., K.M., K.S., R.N., A.K., K.T., Y.Y., H.H., and T. Ohmori; investigation and data analyses, S. Shimojo, A.M., T.M., S. Soth, H.N., M.A.V.R., T.K., K.M., K.S., R.N., S.M., Y.F., T.T., N.B., A.K., Y.K., and Y.Y.; writing – original draft, Y.T.; writing – review and editing, Y.T., T.M., A.K., Y.Y., K.N., Y.H., H.H., T. Ohmori, T. Omasa, and S.U.; supervision, S.U.

## Declaration of interests

M.F., A.M., T.M., M.A.V.R., T.K., K.M., K.S., and K.T. have relationships with U-Medico Inc. that include employment; S.U. has a relationship with U-Medico Inc. as the founder and CSO; R.A. and Y.H. have relationships with Chitose Laboratory Corp. that include employment.

## Supplemental information

Data S1. Table S1

Document S2. Article plus supplemental information
