# Supplementary material for: Manufacture of adeno-associated virus vectors by a novel human-derived cell line HAT and comprehensive evaluation of the vectors
Source: Mol Ther Adv. 2026 Feb 14;34(1):201700. doi: 10.1016/j.omta.2026.201700 (PMC13148930; doi:10.1016/j.omta.2026.201700)
Supplement: Document S1. Figures S1–S14 and Tables S2–S4 [file mmc1.pdf]

OMTA, Volume *34*

Supplemental information

Manufacture of adeno-associated virus vectors

by a novel human-derived cell line HAT

and comprehensive evaluation of the vectors

Yasuo Tsunaka, Mitsuko Fukuhara, Saki Shimojo, Aoba Matsushita, Takahiro Maruno, Sereirath Soth, Haruka Nishiumi, Mark Allen Vergara Rocafort, Toshie Kuwahara, Kenjiroo Matsumoto, Kosei Shibata, Ryoji Nakatsuka, Ryo Asahina, Saho Mizukado, Yuuki Fukai, Tomoki Togashi, Nemekhbayar Baatartsogt, Kimitoshi Takeda, Atsushi Kuno, Yuji Kashiwakura, Yuki Yamaguchi, Kazuaki Nakamura, Yugo Hirai, Hirokazu Hirai, Tsukasa Ohmori, Takeshi Omasa, and Susumu Uchiyama

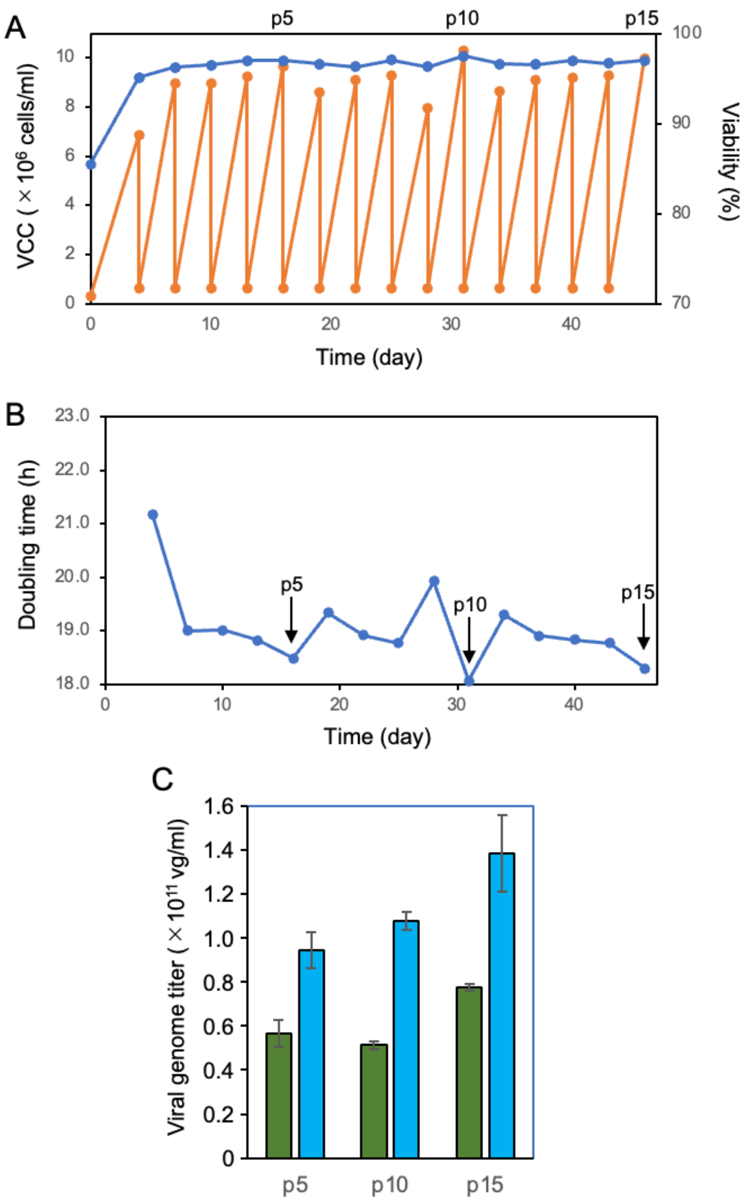

Figure S1. Production of rAAVs at different passages using HAT cells.

(A) Representative profiles of viable cell concentration (VCC; orange line and dots) and cell viability (navy blue line and dots) of HAT cells until passage 15 (p15). (B) Representative profile of doubling time of HAT cells until p15. (C) Bar graphs of virus genome titer of AAV2 (green bar) and AAV9 (cyan bar) produced in HAT cells at p5, p10, and p15. Error bars are ± SD of the mean of triplicate batches.

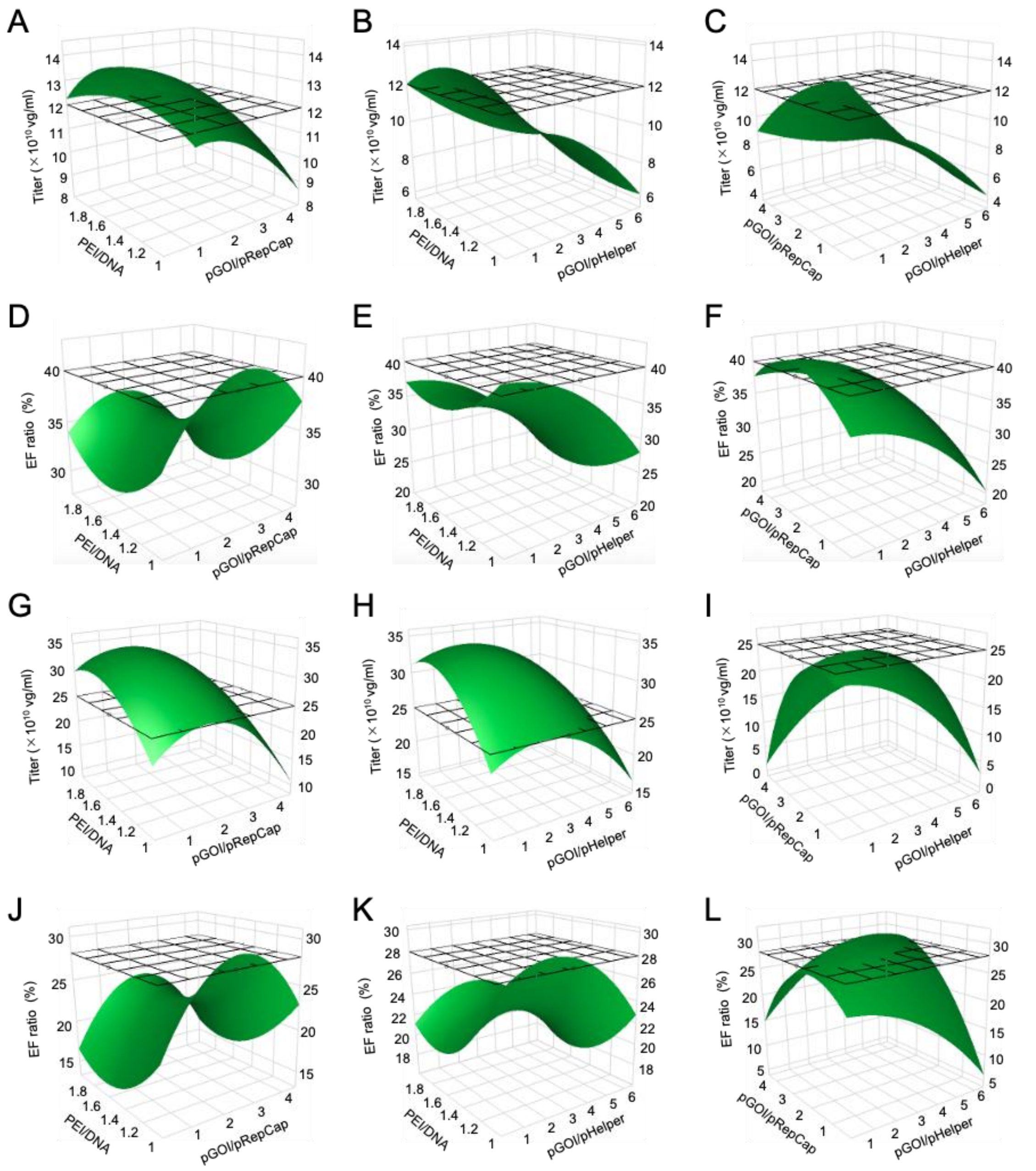

Figure S2. DoE surface three-dimensional (3D) plots of the HAT-cell-produced AAV2 (A–F) and AAV5 (G–L) for titer and the ratios of PEI to DNA and pGOI to pRepCap (A, G), the ratios of PEI to DNA and pGOI to pHelper (B, H), and the ratios of pGOI to pRepCap and pGOI to pHelper (C, I), and for EF ratio and the ratios of PEI to DNA and pGOI to pRepCap (D, J), the ratios of PEI to DNA and pGOI to pHelper (E, K), and the ratios of pGOI to pRepCap and pGOI to pHelper (F, L). Two-dimensional (2D) response grids in the 3D plots are corresponding to the design space plots in Figure 1.

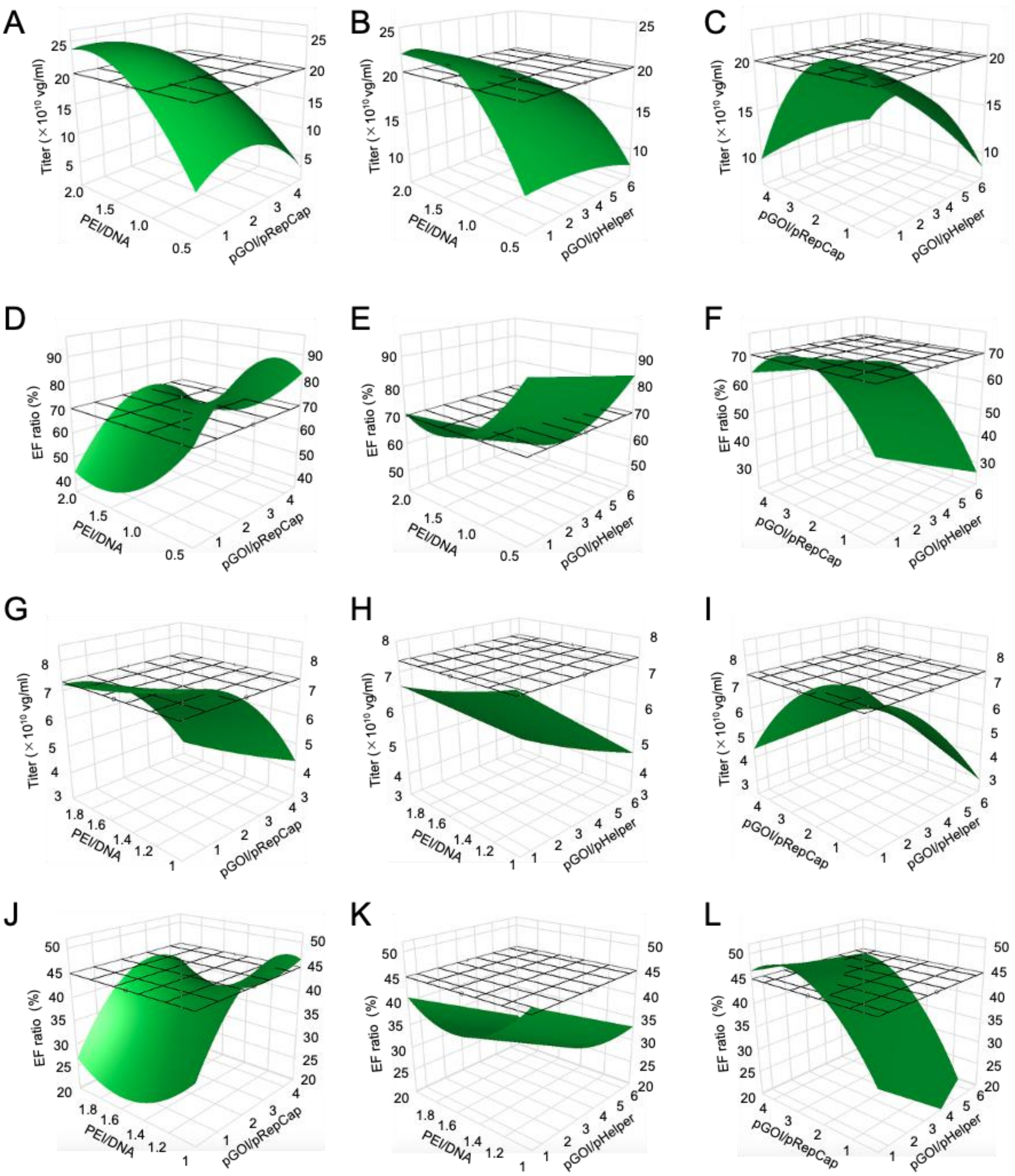

Figure S3. DoE surface 3D plots of the HAT-cell-produced AAV9 (A–F) and the HEK-cell-AAV9 (G–L) for titer and the ratios of PEI to DNA and pGOI to pRepCap (A, G), the ratios of PEI to DNA and pGOI to pHelper (B, H), and the ratios of pGOI to pRepCap and pGOI to pHelper (C, I), and for EF ratio and the ratios of PEI to DNA and pGOI to pRepCap (D, J), the ratios of PEI to DNA and pGOI to pHelper (E, K), and the ratios of pGOI to pRepCap and pGOI to pHelper (F, L). 2D response grids in the 3D plots are corresponding to the design space plots in Figure 1.

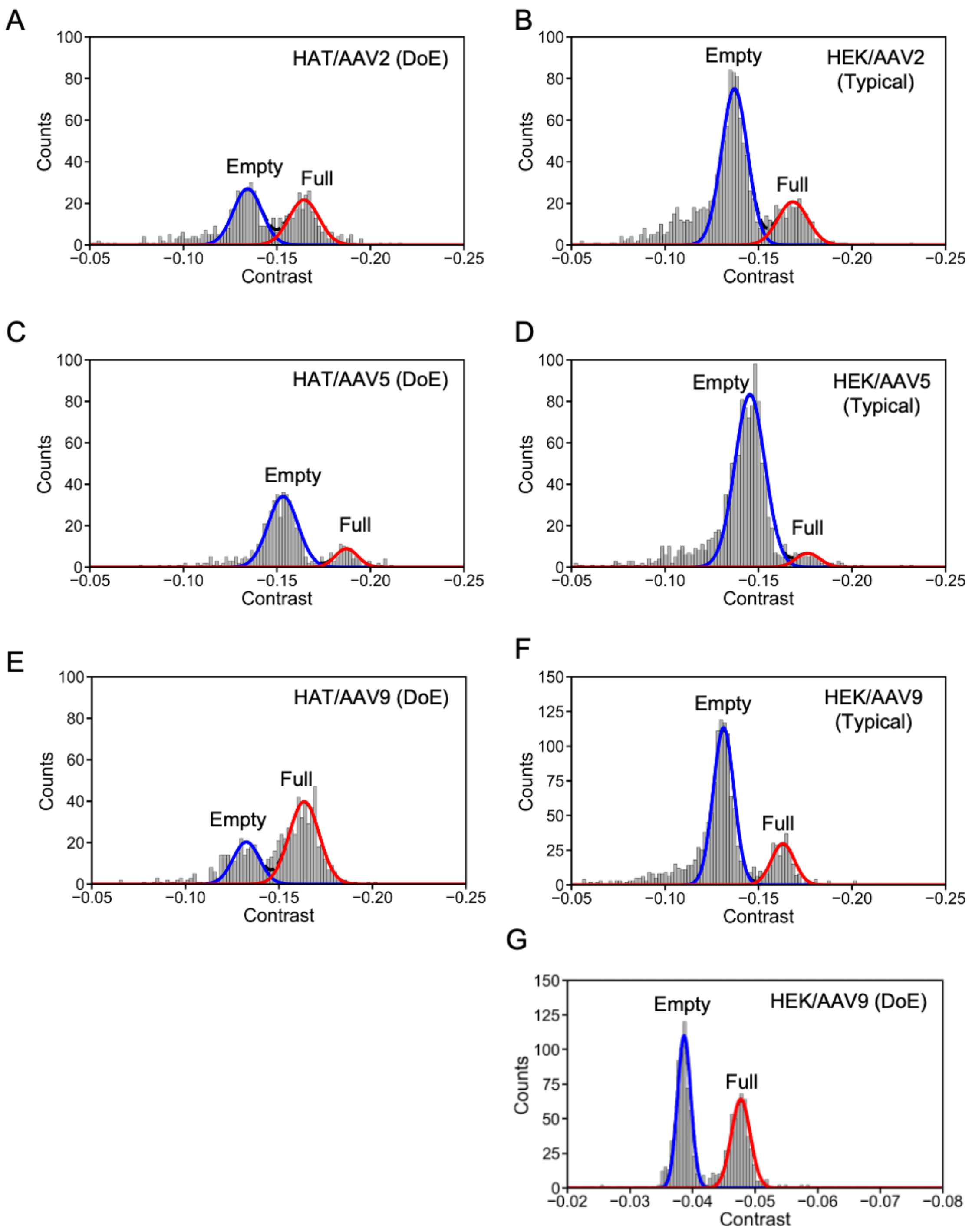

Figure S4. Representative mass photometry histograms of HAT- and HEK-cell-produced rAAV2 (A, B), rAAV5 (C, D), and rAAV9 (E, F, G) after small-scale affinity purification. The HEK-cell-produced rAAV9 shows two histograms under the typical transfection conditions (F) and DoE-optimized conditions (G). The blue and red lines represent the areas of empty particles and full particles, respectively, based on their contrast distributions.

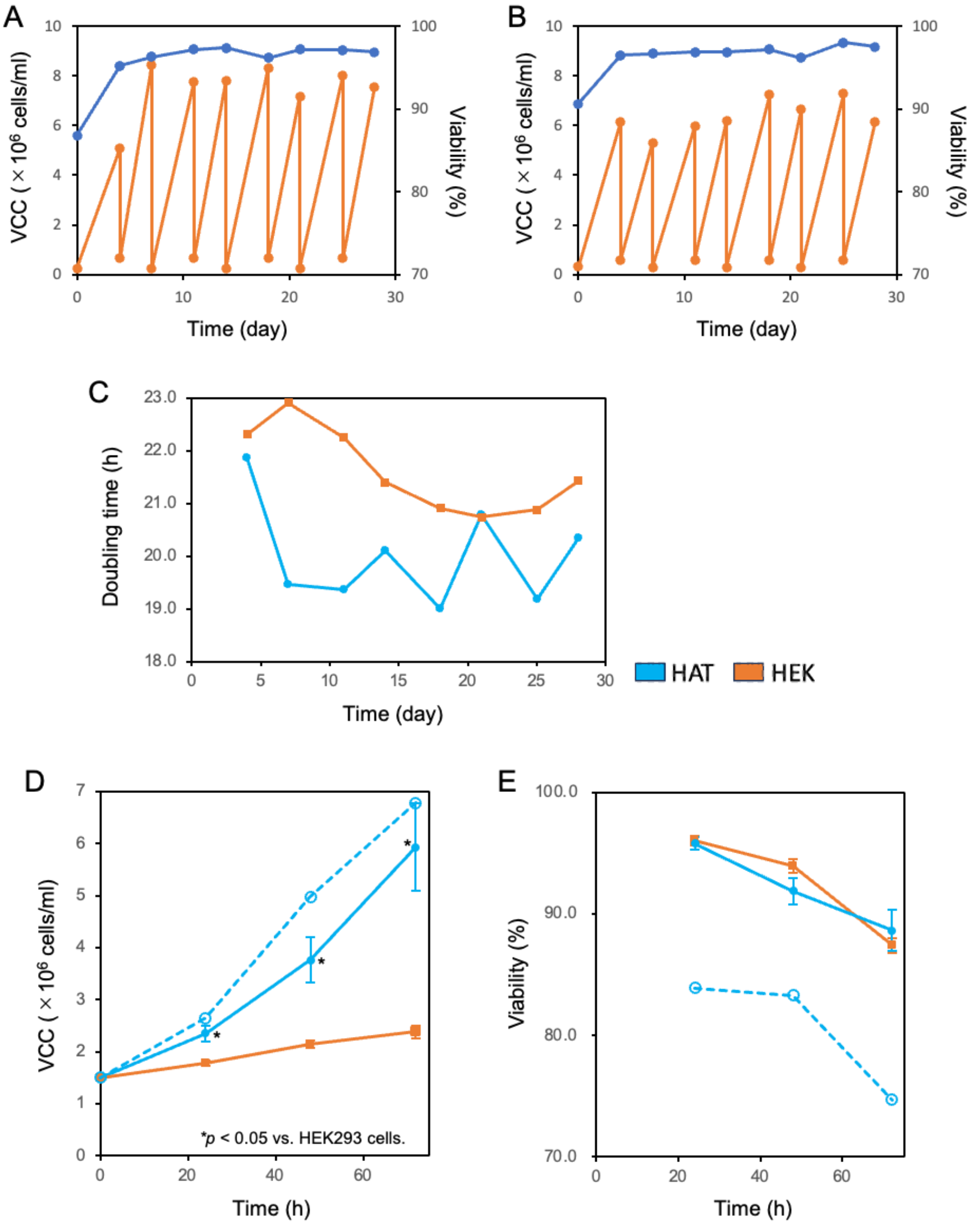

Figure S5. Cell proliferation profiles of HAT cells and HEK293 cells under similar culture condition. (A, B) Profiles of VCC (orange lines and dots) and cell viability (navy blue lines and dots) of HAT cells (A) and HEK293 cells (B) for 28 days under the same culture condition. (C) Profiles of doubling time of HAT cells (cyan line and dots) and HEK293 cells (orange line and dots) for 28 days under the same culture condition. (D, E) Profiles of VCC (D) and cell viability (E) of HAT cells (flask batches, cyan line and cyan closed circle plots; reactor batch, cyan dotted line and cyan open circle plots) and HEK293 cells (orange line and orange square plots) for 72 HPT under each DoE-optimized condition. Error bars are ± SD of the mean of triplicate batches. *$p < 0.05$ *vs.* HEK293 cells.

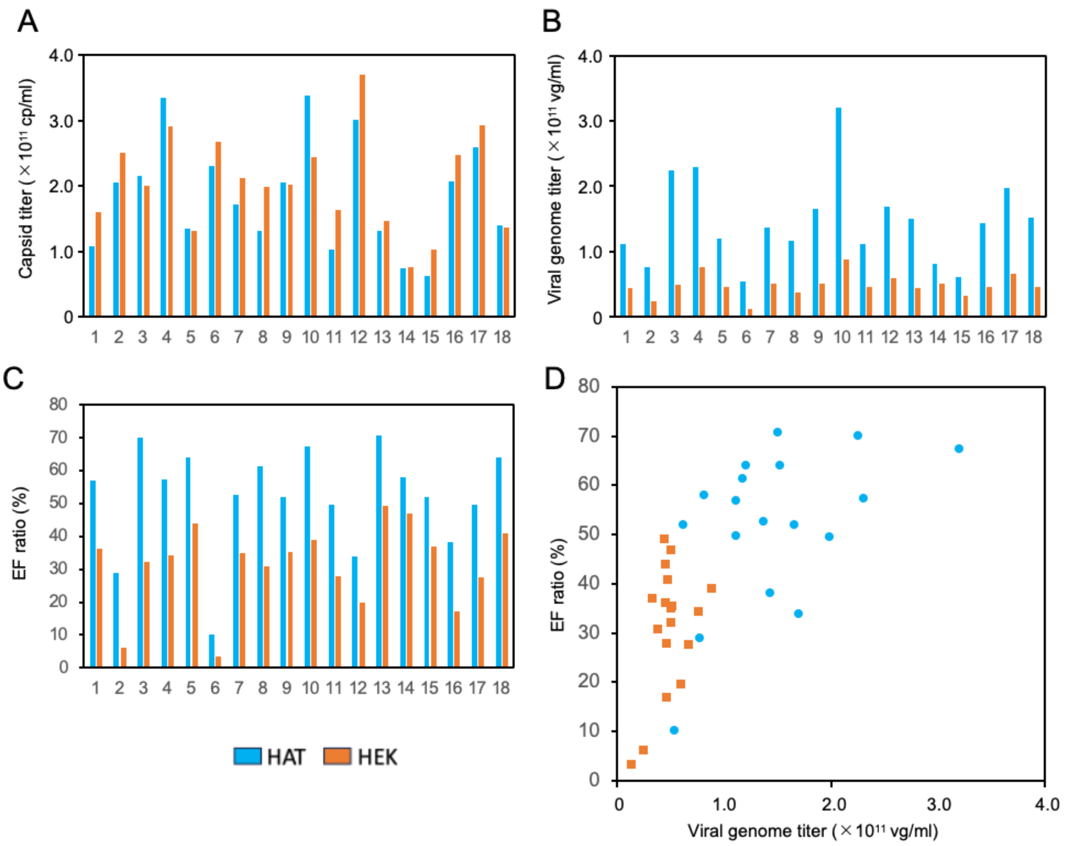

Figure S6. DoE results for rAAV9 produced in HAT and HEK293 cells.

(A–C) Bar graphs showing capsid titer (A), virus genome titer (B), and EF ratio (C) of rAAV9 produced in HAT cells (cyan bars) and HEK293 cells (orange bars) under the same 18 DoE conditions described in Table S1. (D) Scatter plots of virus genome titer versus EF ratio for rAAV9 produced in HAT cells (cyan circles) and HEK293 cells (orange squares) under each DoE experimental condition.

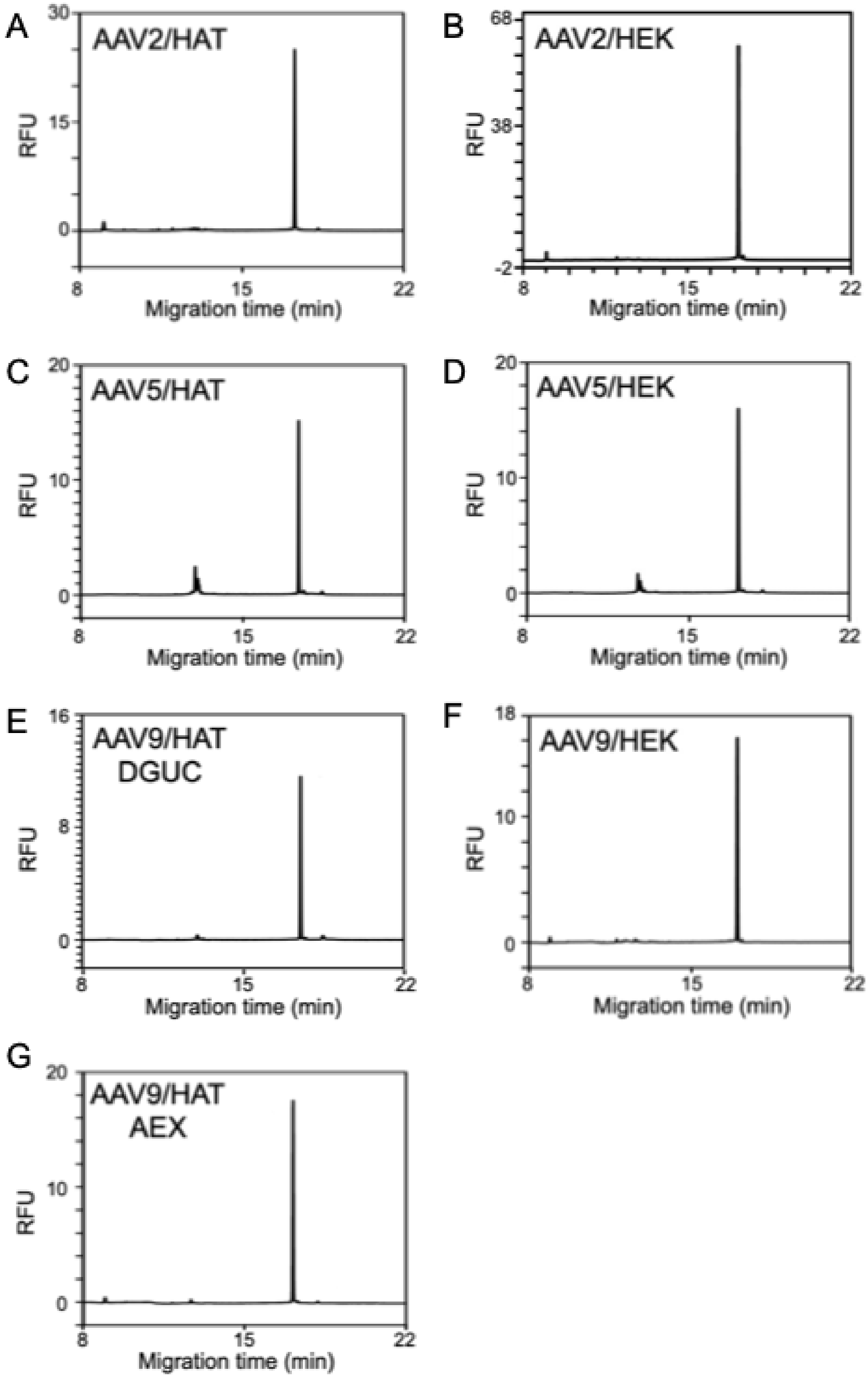

Figure S7. Electrograms of nucleic acid components from the purified rAAV products produced HAT and HEK293 cells.

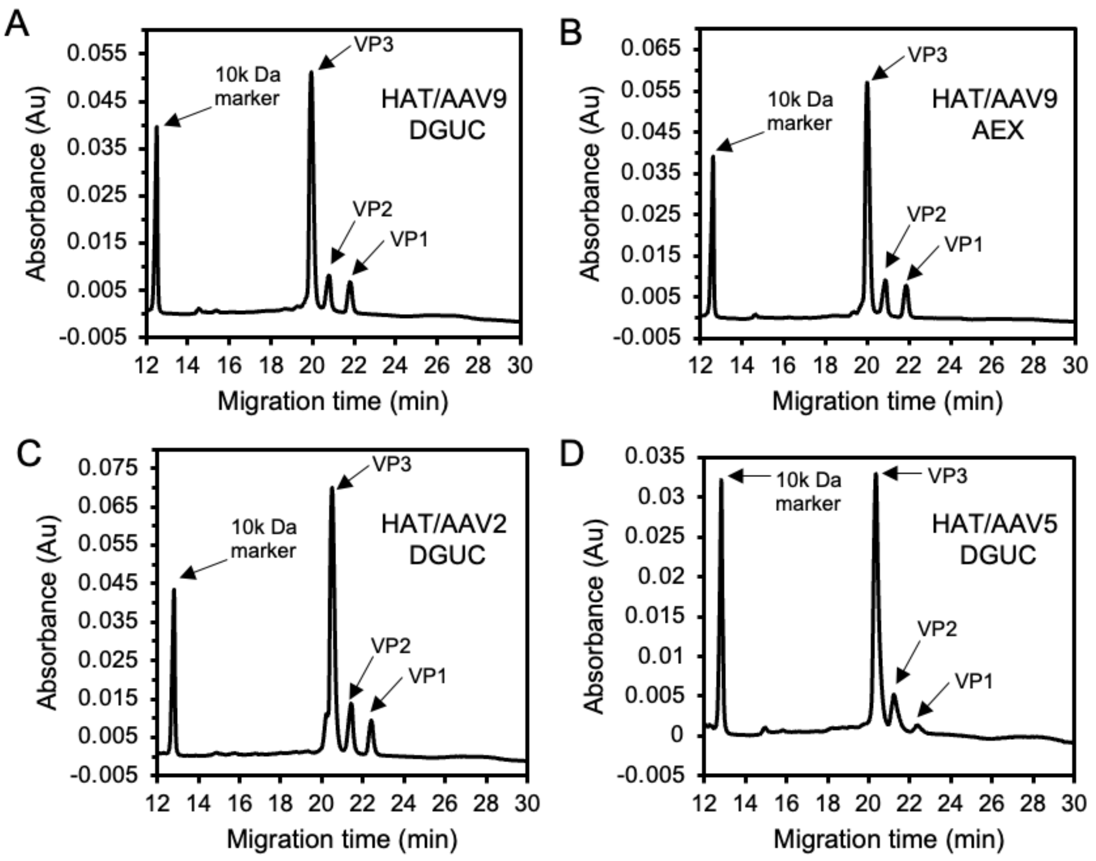

Figure S8. Representative CGE–SDS electropherograms of HAT-cell-produced AAV9 subjected to DGUC (A) or AEX (B) purifications, and HAT-cell-produced AAV2 (C) and AAV5 (D) subjected to DGUC purification.

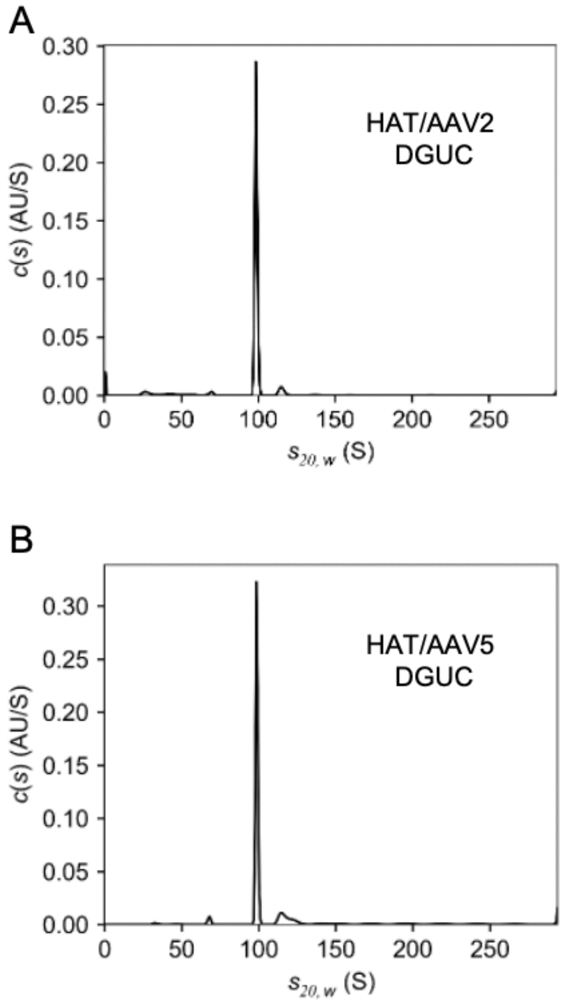

Figure S9. Analytical ultracentrifugation sedimentation profile of HAT-cell-produced AAV2 (A) and AAV5 (B) subjected to DGUC purification.

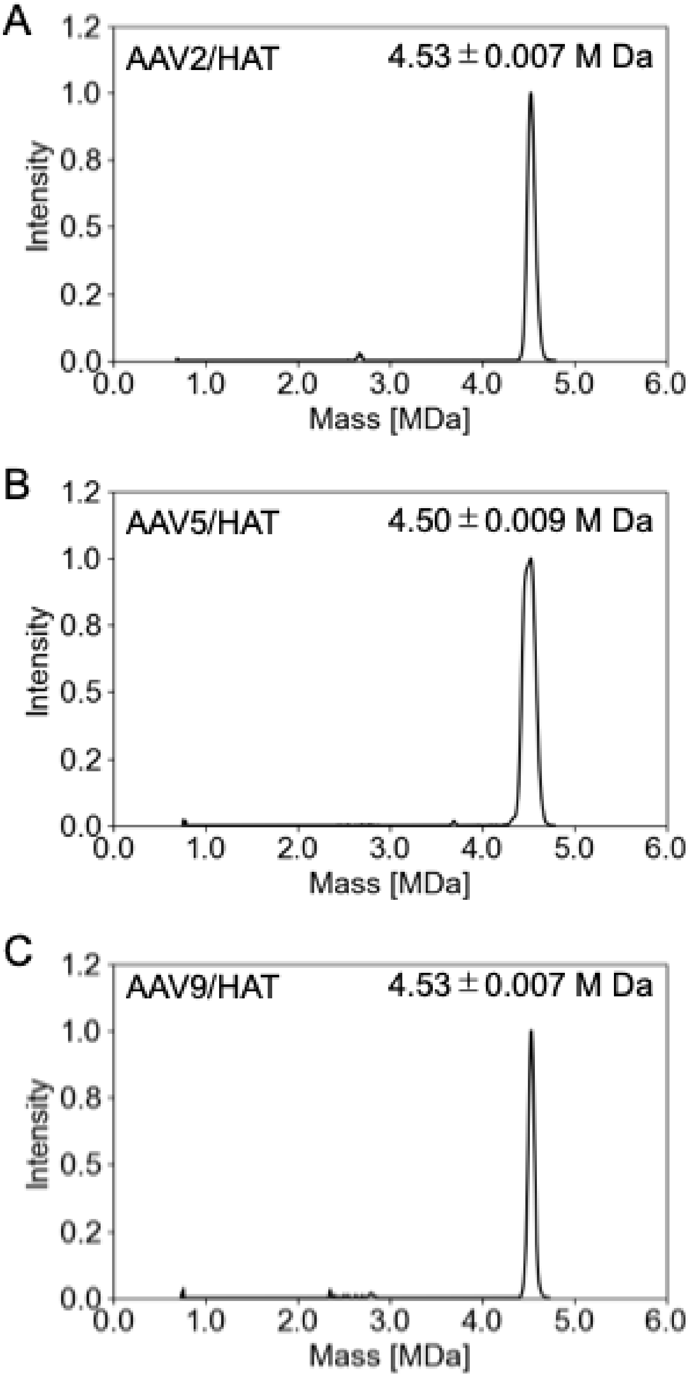

Figure S10. Evaluation of the EF ratio by orbitrap-based charge-detection mass spectrometry (CD–MS). Direct analysis of AAV2 (A), AAV5 (B), and AAV9 (C) using CD–MS. The mass distribution profile verified that the samples was composed entirely of FP (100%), with no detectable EP present. Concurrently, the molecular masses of rAAV2, rAAV5, and rAAV9 were determined to be 4.53 ± 0.01, 4.50 ± 0.01, and 4.53 ± 0.01 MDa, respectively.

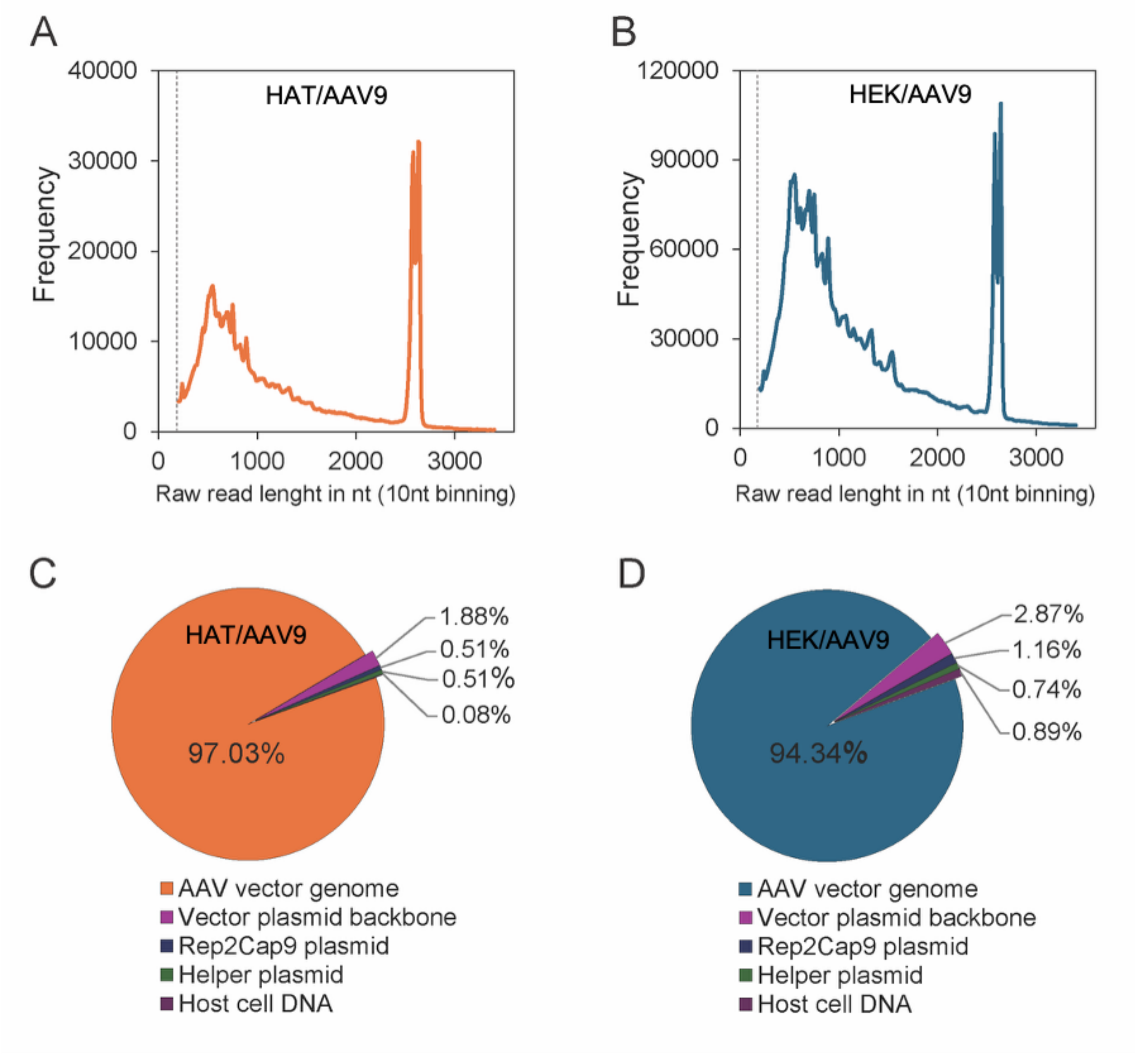

Figure S11.

Size bias in nanopore sequencing reads. (A, B) Size distribution of raw nanopore sequencing reads derived from HAT-cell-produced rAAV9 (A) and HEK-cell-produced rAAV9 (B). (C, D) Mapping distribution of reads aligned to the human genome and plasmid-derived reference sequences in HAT-cell-produced rAAV9 (C) and HEK-cell-produced rAAV9 (D).

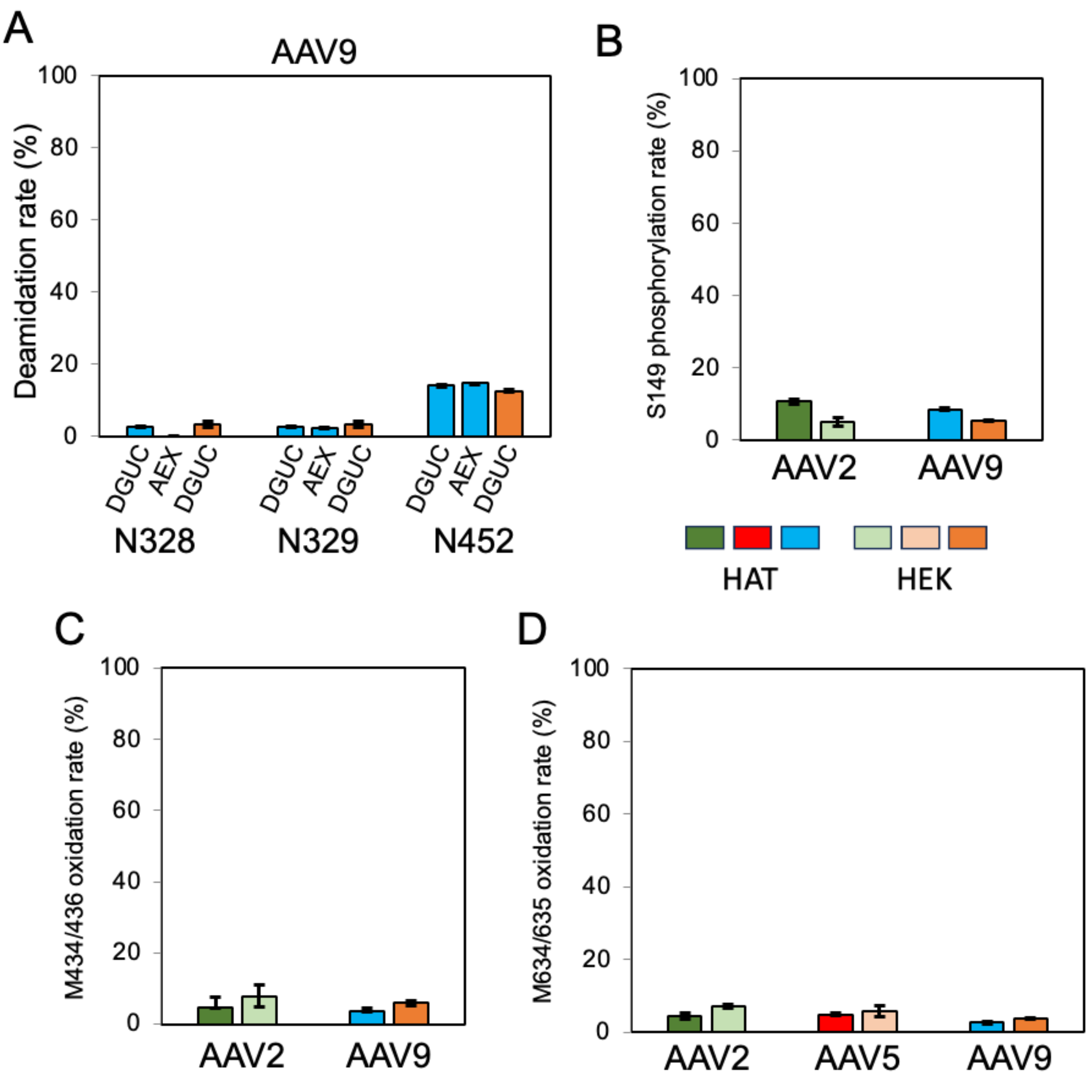

Figure S12. Comparison of post-translational modifications between the HAT- and HEK-cell-derived rAAV products. (A–D) Bar graphs showing the analytical results for the purified products produced by HAT and HEK293 cells in N328, N329, and N452 deamidation (A); S149 phosphorylation (B); M434/436 oxidation (C); and M634/635 oxidation (D). Bars use the same color-coding as in Figure 2. Error bars are ± SD of the mean of triplicate experiments.

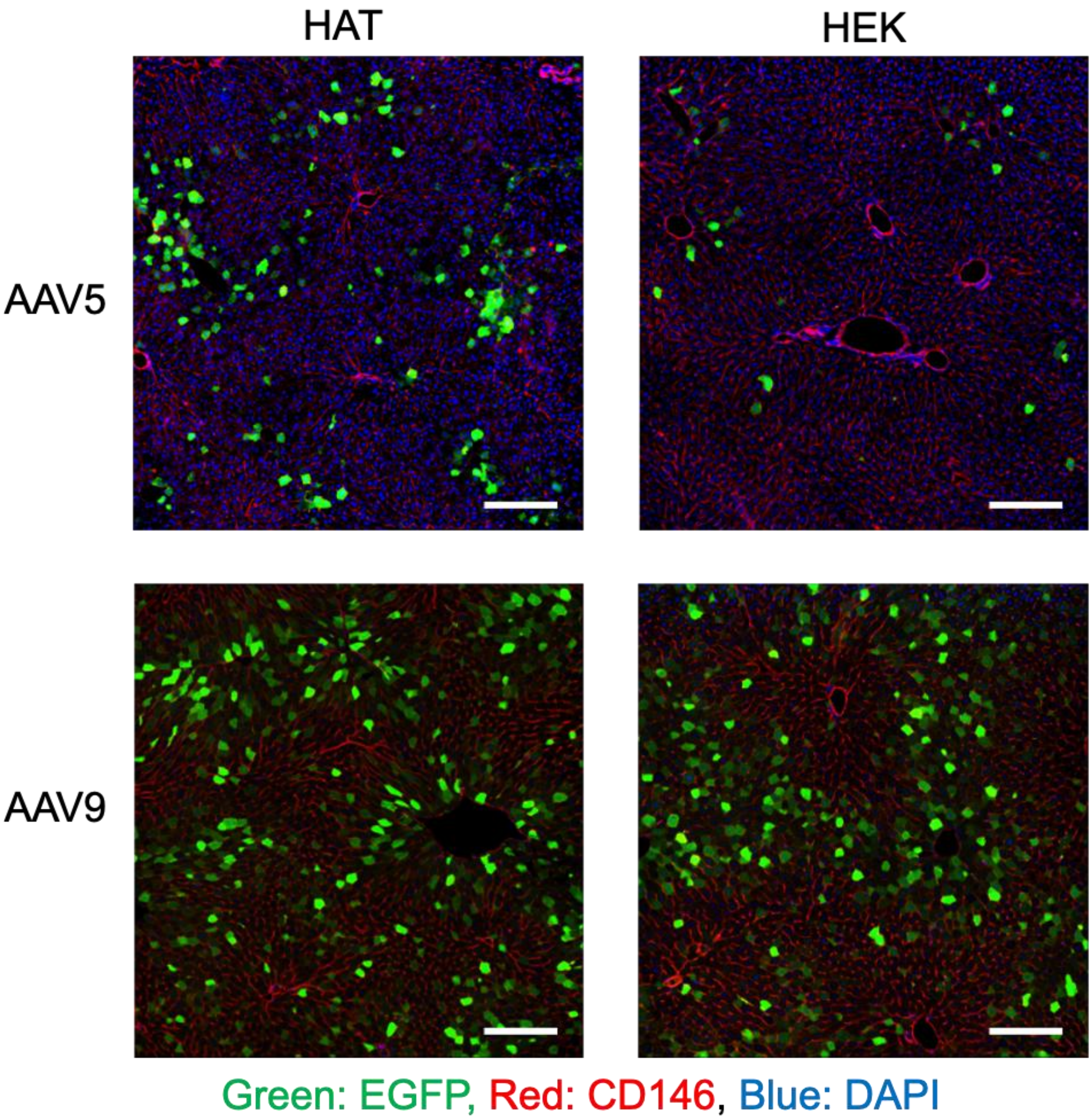

Figure S13. Representative immunostained images in mouse liver at 3 weeks after administration of AAV vectors. EGFP expression in the liver was evaluated by immunostaining (green, EGFP; red, CD146; blue, DAPI). Scale bar, 200 μm.

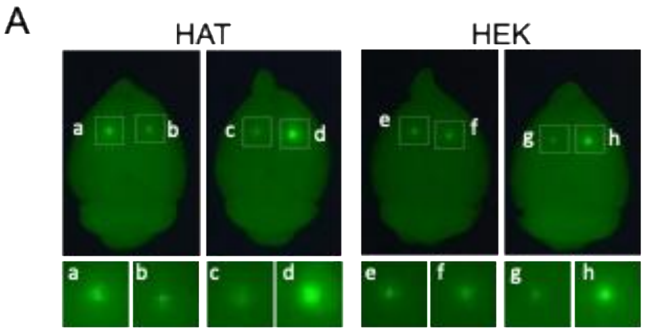

B

Cell type	HAT-derived AAV	HEK-derived AAV
Neuron (NeuN+)	196.3 ± 18.0	203.5 ± 21.5
Astrocyte (GFAP+)	46.3 ± 4.6	51.0 ± 3.9
Microglia (Iba1+)	66.3 ± 8.8	61.3 ± 10.3

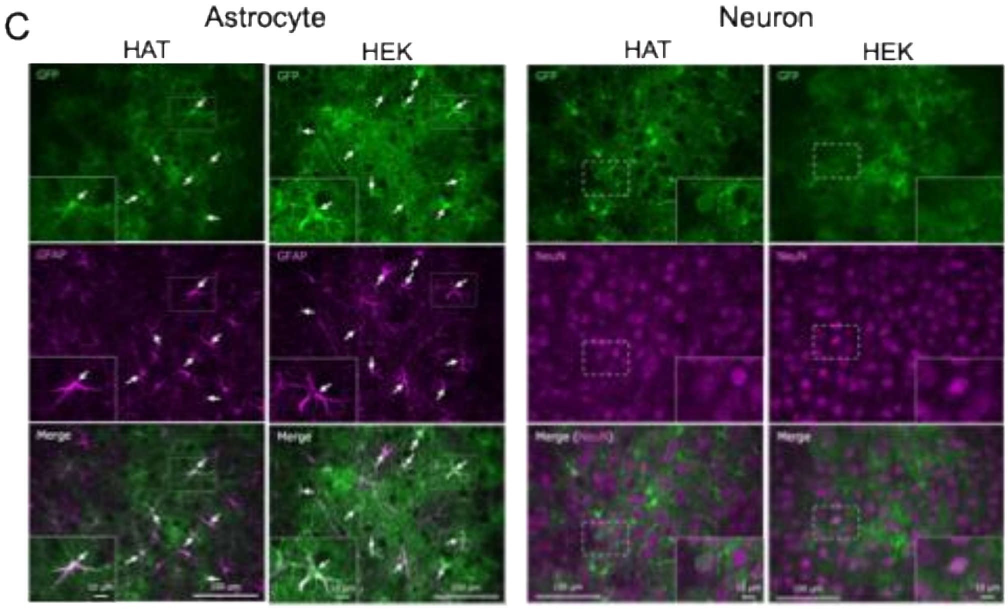

Figure S14. (A) EGFP fluorescence images taken 3 weeks after bilateral AAV injection into the mouse motor cortex. AAV9–CMV–EGFP vectors (2.5×10^{11} vg/mL, 1 μL) produced by HAT (left two panels) or HEK293 cells (right two panels) were injected over 20 min into the primary motor cortex, and mice were perfused 3 weeks later. Enlarged views of the boxed regions (a–h) in the whole-brain images are shown at the bottom of the figure. (B) Comparison of cell densities in the EGFP-positive region. In sagittal sections, neurons, astrocytes, and microglia were counted within EGFP-expressing areas

(313.75 × 313.75 μm²; n = 4 hemispheres). No significant differences were observed between groups. n.s., not significant by unpaired *t*-test. Data are mean ± SD of experimental replicates. (C) Representative immunofluorescence images showing EGFP (green) with GFAP (purple, astrocyte marker, left two columns) or NeuN (purple, neuronal marker, right two columns). Left panels show data of HAT-cell-produced AAV; right panels show data for HEK-cell-produced AAV. Arrows indicate EGFP-expressing GFAP-positive astrocytes or NeuN-positive neurons. Insets in the lower left of each panel show magnified views of the boxed regions with co-labeled cells. Scale bars, 100 μm and 10 μm (insets).

Table S1. DoE Design and Results
Provided as a separate Excel file.

Table S2. VCC and cell viability at harvest of rAAV production

Serotype	Cell	Batch	VCC ($\times 10^6$ cells/mL)	Viability (%)
AAV2	HAT	Flack batch1	8.1	92.0
AAV2	HAT	Flask batch2	7.6	91.5
AAV2	HAT	Flask batch3	8.0	92.2
AAV5	HAT	Flask batch1	10.7	95.3
AAV5	HAT	Flask batch2	10.8	95.5
AAV5	HAT	Flask batch3	10.2	95.8
AAV9	HAT	Flask batch1	9.6	92.5
AAV9	HAT	Flask batch2	10.7	94.7
AAV9	HAT	Flask batch3	10.7	95.1
AAV2	HEK	Flack batch1	2.0	47.2
AAV2	HEK	Flask batch2	2.0	49.2
AAV2	HEK	Flask batch3	2.3	51.1
AAV5	HEK	Flask batch1	4.9	83.2
AAV5	HEK	Flask batch2	4.4	79.6
AAV5	HEK	Flask batch3	3.9	81.5
AAV9	HEK	Flask batch1	2.1	52.0
AAV9	HEK	Flask batch2	2.1	52.1
AAV9	HEK	Flask batch2	1.9	48.0

Table S3. Results of intact MS analyses

Serotype	Capsid protein	Measured mass (Da) *		Theoretical mass (Da)
		HAT cell	HEK293 cell	
AAV2	VP1	81855.61 ± 0.71	81852.96 ± 3.47	81854.90
	VP2	66489.63 ± 1.50	66488.19 ± 2.72	66487.90
	VP3	59973.20 ± 0.32	59973.84 ± 0.39	59973.78
AAV5	VP1	80335.56 ± 0.92	80335.49 ± 0.33	80335.09
	VP2	65282.93 ± 0.29	65282.55 ± 0.11	65282.69
	VP3	59462.49 ± 0.08	59462.17 ± 0.22	59462.37
AAV9	VP1	81291.37 ± 0.45	81292.70 ± 2.63	81290.34
	VP2	66209.55 ± 0.21	66209.39 ± 1.09	66209.76
	VP3	59731.59 ± 0.14	59732.25 ± 0.34	59732.62

* Data are mean ± SD of experimental replicates.

Table S4. DoE-optimized values of the plasmid ratio in the previous studies and this studies

Serotype	Cell	pGOI:	pRepCap:	pHelper	Optimized	Reference
AAV8	HEK	0.31	5	1	Titer	Zhao et al[38]
AAV8	HEK	1	6.1	1.9	Titer & EF ratio	Coplan et al[33]
AAV2	HEK	0.5	3.52	1	Titer & EF ratio	Park et al[40]
AAV9	HEK	0.27	1.44	1	Titer & EF ratio	Park et al[40]
AAV2	HEK	0.5	0.7	0.8	Titer	Tzimou et al[41]
AAV2	HAT	2.1	1	7	Titer & EF ratio	This study
AAV5	HAT	2.3	1.15	1	Titer & EF ratio	This study
AAV9	HAT	2.3	1	4.6	Titer & EF ratio	This study
AAV9	HEK	2	1	4	Titer & EF ratio	This study
